# Software Choice and Sequencing Coverage Can Impact Plastid Genome Assembly–A Case Study in the Narrow Endemic *Calligonum bakuense*

**DOI:** 10.3389/fpls.2022.779830

**Published:** 2022-07-06

**Authors:** Eka Giorgashvili, Katja Reichel, Calvinna Caswara, Vuqar Kerimov, Thomas Borsch, Michael Gruenstaeudl

**Affiliations:** ^1^Systematische Botanik und Pflanzengeographie, Institut für Biologie, Freie Universität Berlin, Berlin, Germany; ^2^Institute of Botany, Azerbaijan National Academy of Sciences (ANAS), Baku, Azerbaijan; ^3^Botanischer Garten und Botanisches Museum Berlin, Freie Universität Berlin, Berlin, Germany

**Keywords:** assembly software, *Calligonum*, genome assembly, plastid genome, phylogenetic position, nucleotide differences, reproducibility, sequencing coverage

## Abstract

Most plastid genome sequences are assembled from short-read whole-genome sequencing data, yet the impact that sequencing coverage and the choice of assembly software can have on the accuracy of the resulting assemblies is poorly understood. In this study, we test the impact of both factors on plastid genome assembly in the threatened and rare endemic shrub *Calligonum bakuense*. We aim to characterize the differences across plastid genome assemblies generated by different assembly software tools and levels of sequencing coverage and to determine if these differences are large enough to affect the phylogenetic position inferred for *C. bakuense* compared to congeners. Four assembly software tools (FastPlast, GetOrganelle, IOGA, and NOVOPlasty) and seven levels of sequencing coverage across the plastid genome (original sequencing depth, 2,000x, 1,000x, 500x, 250x, 100x, and 50x) are compared in our analyses. The resulting assemblies are evaluated with regard to reproducibility, contig number, gene complement, inverted repeat length, and computation time; the impact of sequence differences on phylogenetic reconstruction is assessed. Our results show that software choice can have a considerable impact on the accuracy and reproducibility of plastid genome assembly and that GetOrganelle produces the most consistent assemblies for *C. bakuense*. Moreover, we demonstrate that a sequencing coverage between 500x and 100x can reduce both the sequence variability across assembly contigs and computation time. When comparing the most reliable plastid genome assemblies of *C. bakuense*, a sequence difference in only three nucleotide positions is detected, which is less than the difference potentially introduced through software choice.

## 1. Introduction

The comparative analysis of complete plastid genomes is performed in numerous investigations every year, even though the computational assembly of these genomes has not yet been perfected. Complete plastid genomes constitute a popular information source in various areas of plant evolutionary research, including phylogenetics (e.g., Xu et al., 2019; Koehler et al., 2020), phylogeography (e.g., Moner et al., 2018; del Valle et al., 2019), and population genetics (e.g., Yang et al., 2013; Rogalski et al., 2015). In recent years, the sequencing and comparison of dozens, if not hundreds, of complete plastid genomes per investigation have become commonplace (e.g., Saarela et al., 2018; Huang et al., 2019). Most of these studies generate complete plastid genomes from short-read whole-genome sequencing data (i.e., “genome skimming” data; Bakker, 2017; Twyford and Ness, 2017). Several specialized software tools for the *de novo* assembly of plastid genomes from genome skimming data exist (e.g., Coissac, 2017; Izan et al., 2017; McKain and Wilson, 2017), but the process of generating complete and accurate assemblies from such data remains challenging (Wu et al., 2015; Freudenthal et al., 2020). For example, the use of genome skimming data for plastid genome assembly requires the separation of reads from different genomic compartments of the cell (Twyford and Ness, 2017). If done bioinformatically, this separation is only as accurate as the employed reference genome and its similarity to the target genome (Izan et al., 2017; Jin et al., 2020). Similarly, employing genome skimming data for plastid genome assembly often necessitates the use of read sets that cover the plastid genome with unequal sequencing coverage (Doorduin et al., 2011; Izan et al., 2017). Unequal sequencing coverage runs contrary to the implicit assumption of many genome assembly algorithms that the input reads should cover the target genome homogeneously (Peng et al., 2012; McCorrison et al., 2014; Olson et al., 2019); while primarily observed for the assembly of nuclear genomes, this assumption also seems to be correct for the assembly of plastid genomes (e.g., Stadermann et al., 2015; Soorni et al., 2017). Moreover, the quadripartite structure of most plastid genomes, comprising a long (LSC) and a short (SSC) single-copy region separated by two inverted repeats (IR) (Ruhlman and Jansen, 2014), often requires the manual circularization of linear assembly contigs (Twyford and Ness, 2017) because genome skimming data comprise an amalgamation of different reads, some of which support alternative junction sites (Jin et al., 2020). Furthermore, the direction of the SSC often needs to be homogenized across plastid genomes before their comparison due to the structural heteroplasmy of these genomes (Walker et al., 2015), and genome skimming data typically contain reads representing both configurations (Wang and Lanfear, 2019). Several software tools have been developed to accommodate some of these challenges (e.g., Ankenbrand et al., 2018; Carrion et al., 2020; Wu et al., 2021), but the process of plastid genome assembly from genome skimming data remains imperfect.

The choice of assembly software and the depth of sequencing coverage have been highlighted as potential sources for low assembly quality among plastid genomes, but a characterization of their impact has yet to be conducted. Several recent investigations have reported factors that may influence the accuracy of plastid genome assembly, including software choice (Freudenthal et al., 2020) and sequencing coverage (reviewed in Gruenstaeudl and Jenke, 2020). The choice of assembly software has been reported as a source of inconsistency in genome assembly by several previous studies (e.g., Magoc et al., 2013; Morrison et al., 2014). In the *de novo* assembly of plastid genomes from genome skimming data, such inconsistency may be associated with differences between assembly algorithms: while some software tools have implemented algorithms that conduct a cyclical sequence extension from a single “seed” sequence (e.g., Dierckxsens et al., 2017), others employ a kmer-based construction of contigs, followed by the concatenation of multiple contigs based on sequence overlap and similarity to a reference genome (e.g., Bakker et al., 2016; McKain and Wilson, 2017). Accordingly, Freudenthal et al. (2020) found considerable differences among the results of different assembly software despite employing the same input sequence data. Interestingly, many of the assembly differences identified by Freudenthal et al. (2020) corresponded to competing locations or orientations of the four plastid genome regions rather than nucleotide polymorphisms. The question if alternative plastid genome assemblies generated for the same taxon could impact downstream analyses such as species identification or phylogenetic inference has so far not been addressed.

Differences in sequencing coverage have also been reported as a source for distinct plastid genome assemblies. Doorduin et al. (2011), for example, found that the number of SNPs across the plastid genomes of multiple individuals of *Jacobaea vulgaris* varied between different regions of the genome depending on the depth of sequencing coverage. Similarly, Kim et al. (2015) reported a correlation between cases of local misassembly and regions with exceptionally high sequencing coverage in plastid genomes of rice; regions of exceptionally high coverage are often associated with genome skimming data (Twyford and Ness, 2017). Moreover, Izan et al. (2017) found that regions with low sequencing coverage were not correctly assembled under default software settings in several angiosperm plastid genomes. Indeed, genome assemblies with unequal sequencing coverage are often characterized by high rates of sequencing error (Hubisz et al., 2011). Based on these observations, some assembly pipelines pre-select sequence reads that represent a low but comparatively even sequencing coverage for genome assembly (e.g., 20x; Soorni et al., 2017), and different studies indicated that a sequencing coverage of 30–50x is needed at a minimum for reliable plastid genome assembly (e.g., Soorni et al., 2017; Twyford and Ness, 2017; Sharpe et al., 2020). Sequencing coverage has, thus, been identified as an important indicator of assembly quality, especially in plastid genomes (Gruenstaeudl and Jenke, 2020). Gu et al. (2016), for example, employed sequencing coverage as an indicator to refine the assembly of the plastid genome of *Lagerstroemia fauriei*. Despite the importance of sequencing coverage for the successful assembly of plastid genomes, few, if any, studies have aimed to characterize the resulting assembly differences or evaluated if those differences are large enough to impact downstream analyses.

In this study, we test the impact of software choice and sequencing coverage on the process of plastid genome assembly in a species for which a correct assembly is vital for conservation efforts. Specifically, we use the threatened and narrow endemic shrub *Calligonum bakuense* (Polygonaceae) as a test case for evaluating the variability in plastid genome assembly caused by software choice and levels of sequencing coverage. The entire species comprises only 170–200 individuals which are currently inhabiting approximately seven localities around the Absheron Peninsula near Baku, the capital city of the Republic of Azerbaijan. *Calligonum bakuense* represents an exemplary case where a precise assembly of the plastid genome is of great importance to delineate the species, determine its correct phylogenetic placement relative to other members of the genus, and assess its genetic diversity at the population level. Genomic information on *C. bakuense* is currently absent, and documenting its complete plastid genome would be an important asset for future investigations on this rare and declining species. In this study, we use genome skimming data from two individuals of *C. bakuense* to characterize differences across genome assemblies in response to the choice of assembly software and levels of sequencing coverage. Specifically, we test whether the plastid genome assembly of *C. bakuense* is consistent across four commonly employed assembly software tools and seven different levels of sequencing coverage, and if any differences among the resulting assemblies can potentially affect the outcome of phylogenetic tree reconstruction. Based on our findings, we discuss the consequences that differences in plastid genome assembly of the magnitude detected here could have on biological conclusions and we make recommendations to optimize the assembly of complete plastid genomes.

## 2. Materials and Methods

### 2.1. Biology and Distribution of *Calligonum bakuense*

**Figure 1:**
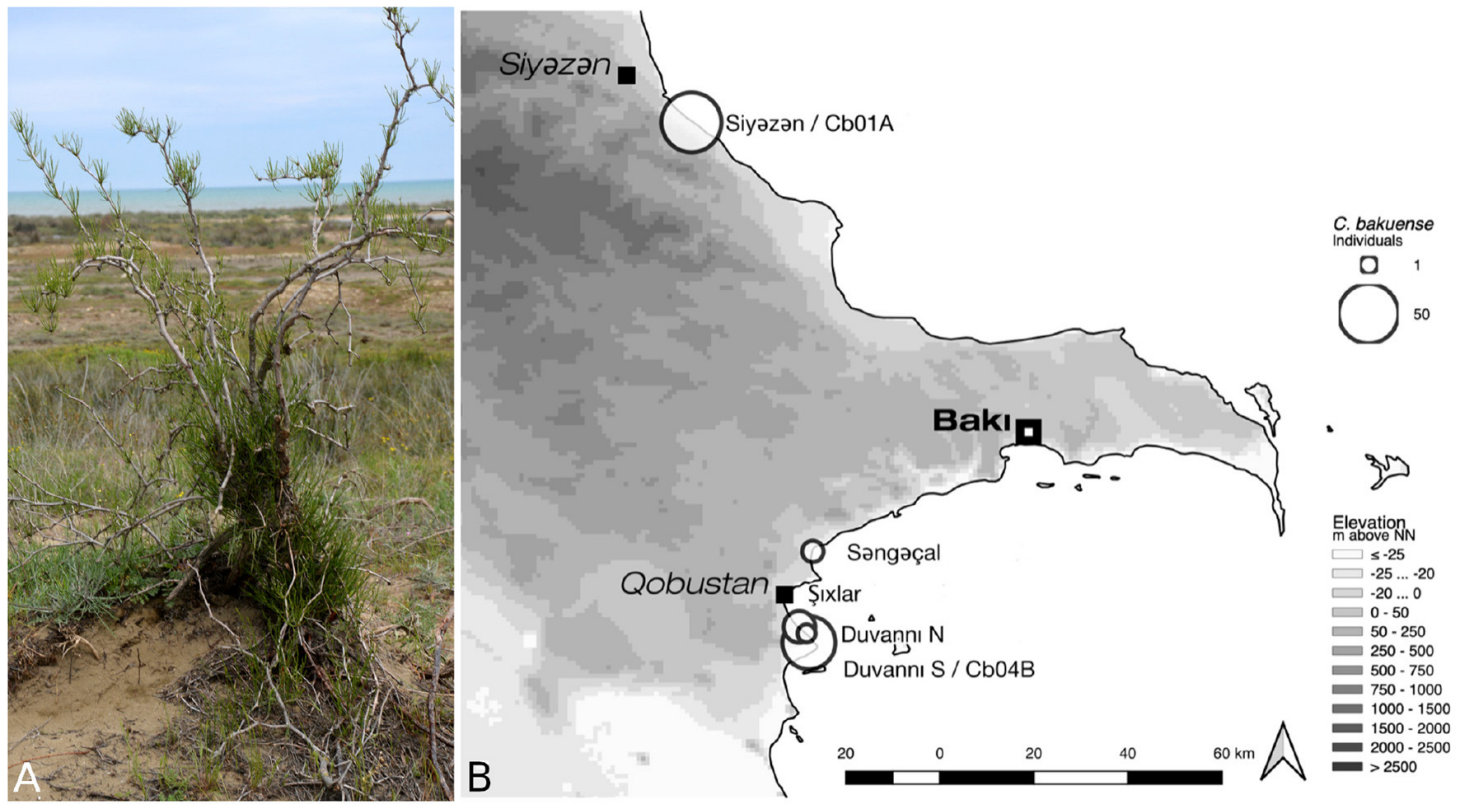
Habit and natural environment **(A)** and the current distribution area **(B)** of *C. bakuense*. The map indicates the localities of all sampled natural populations of *C. bakuense*, including those that individuals Cb01A and Cb04B were sampled from.

*Calligonum bakuense* LITV. is a psammophytic shrub endemic to coastal sand dune areas along the western Caspian shoreline near the city of Baku (Karjagin, 1952; Soskov and Akhmed-Zade, 1974). The species is a unique and declining element of the flora of Azerbaijan and of high conservation interest (Atamov, 2008). It currently comprises a total of seven wild populations that are distributed across a distance of approximately 120 km and collectively contain roughly 170–200 individuals (Figure 1). Here we assemble and report the plastid genomes of two individuals that represent the northern- and the southernmost localities of the current distribution area. The evolutionary relationships of *C. bakuense* to other members of *Calligonum* are currently unknown, as is the population structure within the species. *Calligonum* L. is a lineage of xerophytic shrubs with an estimated 30–40 species; it is distributed from northern Africa, the Arab Peninsula, South West Asia, the Caucasus, the Irano-Turanian region, and Central Asia to China (Brandbyge, 1993; Abdellaoui et al., 2011). Several species of the genus are globally red-listed and exhibit declining population sizes (Baillie et al., 2004). Currently, there is no comprehensive molecular phylogeny of *Calligonum*, but complete plastid genome sequences have been shown as a promising basis for inferring phylogenetic relationships among Chinese members of the genus (Song et al., 2020).

### 2.2. DNA Extraction and Genome Skimming

For a conservation genetic study on *C. bakuense*, silica-dried tissue samples from all known individuals of the species were collected between 2013 and 2015. One individual (Cb01A) that represents the northernmost and one (Cb04B) that represents the southernmost locality of the current distribution area were selected for low-coverage whole-genome sequencing (Figure 1). Whole-genomic DNA of each individual was extracted using a modified CTAB protocol (Borsch et al., 2003), sheared via ultrasonication to an average fragment size of ~300 bp, and converted to a barcoded genomic library using the Illumina TruSeq DNA sample preparation kit (Illumina, San Diego, CA, USA) under the high sample protocol of the manufacturer. The DNA of both individuals was pooled equimolarly and sequenced on a full Illumina HiSeq 4000 plate by Macrogen Inc. (Seoul, South Korea). If evenly distributed, this amount of sequence data would cover the nuclear genome (1C) of each individual with an average sequencing coverage of 18–20x. After sequencing, low-quality bases (phred-score <20) and remnants of Illumina adapter sequences were trimmed from the raw reads with Cutadapt v. 1.14 (Martin, 2011). While primarily intended for the development of genetic markers in the nuclear genome, this sequence data also comprises a high number of reads representing the plastid genome, rendering the data ideal to evaluate the impact of software choice and the depth of sequencing coverage on plastid genome assembly.

### 2.3. Computational Extraction of Plastid Genome Reads

Paired sequence reads of the plastid genome were bioinformatically extracted from the raw sequence data as input for plastid genome assembly. This extraction was primarily conducted due to the large number of raw sequence reads generated, which exceeded the maximum capacity of some of the assembly software tools employed (e.g., IOGA terminates with a memory error when operating on the raw sequence data). Hence, we mapped the raw sequence reads to a set of related, previously published plastid genomes and then extracted and retained only the successfully mapped, paired reads using script 5 of the pipeline described in Gruenstaeudl et al. (2018). Since the phylogenetic position of *C. bakuense* has not yet been evaluated on a molecular basis, we selected a taxonomically broad set of twelve plastid genomes of the Caryophyllales as reference genomes: *Fagopyrum esculentum* subsp. *ancestrale* (GenBank accession number NC_010776), *Fallopia multiflora* (NC_041239), *Rumex acetosa* (NC_042390), *Muehlenbeckia australis* (MG604297), *Oxyria sinensis* (NC_032031), and *Rheum palmatum* (NC_027728, all Polygonaceae); *Amaranthus hypochondriacus* (NC_030770) and *Chenopodium quinoa* (NC_034949, both Amaranthaceae); *Mesembryanthemum crystallinum* (NC_029049, Aizoaceae); *Carnegiea gigantea* (NC_027618, Cactaceae); *Dianthus caryophyllus* (NC_039650, Caryophyllaceae); and *Nyctaginia capitata* (NC_041415, Nyctaginaceae).

### 2.4. Capping of Sequencing Coverage

To determine if the process of plastid genome assembly is consistent across different levels of sequencing coverage, we created subsets of the plastid genome read set with a lower average sequencing coverage (hereafter “capped read sets”). Following Sims et al. (2014), we use the term "sequencing depth" to specifically denote the average sequencing coverage of a genome or genome region hereafter. We capped the sequencing coverage of the plastid genome at six different levels, which collectively represent the range of sequencing depths typically encountered in genome skimming: 2,000x, 1,000x, 500x, 250x, 100x, and 50x. The largest evaluated cap level of sequencing coverage (i.e., 2,000x) represents approximately 25% of the uncapped sequencing depth of the plastid genome and indicates roughly the maximum input capacity of the assembly software tool (i.e., IOGA) that was found to require the largest amount of primary memory for the plastid genome assembly of *C. bakuense*. The smallest evaluated cap level of sequencing coverage (i.e., 50x) represents less than 1% of the uncapped sequencing depth of the plastid genome and is located between the minimum and the desirable sequencing coverage for plastid genome sequencing according to Twyford and Ness (2017). Bioinformatically, the cap in sequencing coverage was not a hard threshold above which all additional reads were removed, but a soft threshold above which additional reads were progressively curtailed. In contrast to a hard cap, such a soft cap of sequencing coverage generates a coverage distribution similar to that of empirical sequence data. For example, under a soft cap of 1,000x, some nucleotides of the target genome are supported by more than 1,000 mapped reads and some by less, whereas under a hard cap of 1,000x, none of the nucleotides of the target genome are supported by more than 1,000 mapped reads but some still by less. Technically, this soft cap was implemented through a one-tailed normalization of the sequencing coverage using the script 'bbnorm.sh' of the software BBtools v.33.89 (Bushnell, 2015) under default settings and using the plastid genome of *Calligonum caput-medusae* (MN202600; Song et al., 2020) as a structural reference. All capped read sets were treated identically to the uncapped read set during the genome assembly and all subsequent analyses. For greater efficiency, the read sets capped at 2,000x and 500x were employed as representatives for all six cap levels in the evaluation of the suboptimal assembly software tools FastPlast and IOGA as well as the impact of seed sequence selection on plastid genome assembly.

### 2.5. Genome Assembly

To determine if the process of plastid genome assembly for *C. bakuense* is consistent across different assembly software tools, we compared the assembly results of four commonly-used tools: NOVOPlasty v.3.8.3 (Dierckxsens et al., 2017), GetOrganelle v.1.6.4 (Jin et al., 2020), FastPlast v.1.2.8 (McKain and Wilson, 2017), and IOGA v.38.26 (Bakker et al., 2016). Each of these software tools had been designed for the *de novo* assembly of plastid genomes from short sequence reads and had demonstrated its utility in previous plastid genomic studies (reviewed in Freudenthal et al., 2020). To improve the comparability of the assembly process across these tools, we employed each software under its default settings. To ensure a uniform software execution and to compare computation times across the tools, all assemblies of *C. bakuense* were conducted on the high-performance computer cluster 'Curta' of the Freie Universität Berlin under the following settings: a single 64-bit processor, an allotment of 2 GB of RAM, and a disk I/O speed of 129 MB/s. The raw output, as well as the log file of each assembly run, are available on Zenodo under https://zenodo.org/record/6577786.

In practice, plastid genome assembly software often generates multiple incomplete, linear contigs instead of one complete, circular genome sequence (Twyford and Ness, 2017). Incomplete contigs typically require manual intervention to be combined into a complete genome sequence (Gruenstaeudl et al., 2018). In this study, two of the software tools produced incomplete contigs for *C. bakuense*. In such cases, we concatenated the incomplete contigs upon removing any end overhangs, followed by circularization of the resulting super-contig. The concatenation of contigs was conducted by hand in Geneious v.11.1.4 (Kearse et al., 2012) through aligning each contig to the structural reference genome (*C. caput-medusae*) and then sorting the contigs according to their relative position. If adjacent contigs overlapped for at least 15 bp without differences in their nucleotide sequence, they were merged into a larger contig until all such contigs were combined into a single super-contig.

The identification of the endpoint of a circular genome sequence is challenging for most genome assembly algorithms (but see Wu et al., 2021) and often results in the detection of different endpoints across tools. To avoid inflating the number of differences between assemblies due to unequal endpoints, we manually corrected super-contigs if the inferred endpoints were within 100 bp across assemblies. Specifically, we searched for the first and the last 25 bp of the super-contig of each assembly via separate motif searches, with the maximum number of mismatches set to 3 bp. Any matches within 100 bp of the super-contig ends were considered to be instances where the assembly process extended the sequence beyond its actual endpoint. Such sequence motifs were removed from one of the two ends, followed by circularization of the super-contig. Similarly, poly-N motifs in contigs are often generated by plastid genome assembly software to indicate areas of sequence uncertainty. To avoid inflating the number of differences between assemblies, we automatically corrected poly-N-motifs using the software Pilon v.1.23 (Walker et al., 2014). Moreover, most software tools for plastid genome assembly do not automatically standardize the orientation of the SSC across assemblies, even though plastid genome isomers with alternative SSC orientations naturally exist in most land plants (Walker et al., 2015). To avoid inflating the number of differences between assemblies, we manually homogenized the orientation of the SSC across assemblies using Geneious.

### 2.6. Replication of Assembly Runs

Several software tools for plastid genome assembly constitute multi-step pipelines rather than single applications (Gruenstaeudl et al., 2018). These pipelines typically employ a third-party assembly tool as their core assembly engine to conduct a k-mer-based alignment of reads for the inference of de Bruijn graphs (Izan et al., 2017). FastPlast, IOGA, and GetOrganelle, for example, utilize the assembly software SPAdes (Bankevich et al., 2012) as core assembler, even though the full reproducibility of bacterial genome assemblies with SPAdes has been called into question (e.g., Liao et al., 2015; Souvorov et al., 2018). To characterize potential occurrences of spurious, non-reproducible inferences of de Bruijn graphs, we conducted every plastid genome assembly of the uncapped read set twice under the same input data and software settings (i.e., replicate run #1 and #2). The comparison of these replicate runs allowed us to ascertain the baseline replicability of plastid genome assemblies under these assembly tools.

The cyclical sequence extension that starts from a single “seed” sequence and is implemented in several assembly algorithms (e.g., Dierckxsens et al., 2017) may represent a source of contig variability not present in other assembly algorithms. Several studies have reported minor differences in the number and sequence of assembly contigs depending on the precise seed sequence employed and have, therefore, attempted to identify universally applicable seed sequences (e.g., Lim et al., 2018; Wu et al., 2021). To ensure that seed selection did not inflate the number of differences between assemblies, we employed the same seed sequence for each plastid genome assembly with NOVOPlasty. Moreover, we evaluated if seed selection represented a relevant source of contig variability in our dataset by repeating plastid genome assembly with NOVOPlasty and the 2,000x and 500x capped read sets under a second seed sequence. Both seeds (i.e., seed #1 and seed #2) were arbitrarily selected from the read set.

### 2.7. Sequence Annotation

To enable consistent sequence annotations across all plastid genome assemblies of *C. bakuense*, the sequence annotations from two existing plastid genomes of *Calligonum* were transferred to the new assemblies using Geneious. Specifically, we automatically transferred all gene, tRNA, and rRNA annotations from the plastid genomes of *C. caput-medusae* and *C. arborescens* (MN202599; both Song et al., 2020) to the assemblies of *C. bakuense* based on a sequence similarity threshold of 95%. Upon transfer, we conducted a manual inspection of the transferred annotations for each coding region regarding the presence of start and stop codons, the absence of internal stop codons, and their lengths as a multiple of three. Any premature stop codon that was introduced by the transfer process but not based on the nucleotide sequence was corrected; any premature stop codon based on the nucleotide sequence was recorded as an indicator of low assembly quality. The annotations of the IRs and, by extension, of the single-copy regions were inferred for each assembly using script 4 of the pipeline of Gruenstaeudl et al. (2018).

### 2.8. Evaluation of Assembly Quality

To assess the quality of the plastid genome assemblies of *C. bakuense* and, simultaneously, the performance of each assembly software, the raw output of each assembly process was evaluated with Quast v.4.6.3 (Gurevich et al., 2013). Specifically, we assessed and compared the number, length, and contiguity of the contigs generated by each assembly software. NGA50 and LGA50 were calculated as contiguity metrics (Earl et al., 2011; Gurevich et al., 2013). As part of this quality assessment, we also compared the computation times of the different assembly software tools employing different read sets. All assembly statistics were calculated after the removal of contigs smaller than 100 bp, if any, to avoid the counting of mono- or di-nucleotide fragments.

### 2.9. Characterization of Assembly Differences

To compare the different plastid genome assemblies of *C. bakuense* as generated by different software tools, levels of sequencing coverage, seed selection, and run replication, we conducted a series of statistical evaluations based on pairwise genetic distances. As the basis for these comparisons, we generated pairwise alignments of the assemblies using MAFFT v.7.471 (Katoh and Standley, 2013) under default settings. We then inferred the differences in sequence as well as in length of the four genome regions (i.e., LSCs, IRb, SSCs, and IRa) for each plastid genome pair. Sequence differences were calculated as the number of single nucleotide polymorphisms (SNPs) when excluding gaps but including nucleotide ambiguities using trimAl v.1.2 (Capella-Gutierrez et al., 2009). Length differences were calculated as the absolute difference across the lengths of different genomic regions; this metric is independent of the exact number of regions per genome but may overestimate similarity, as dissenting length changes across regions may compensate each other. Upon calculation, difference values were aggregated in a pairwise genetic distance matrix. Since only the plastid genomes assembled with GetOrganelle using the capped read sets of 500x, 250x, and 100x were identical within both samples and across all tested parameters and, thus, best-supported, we designated the assembly inferred with GetOrganelle on the 500x capped read set as the "final" plastid genome sequence for each individual of *C. bakuense*. To verify the length and sequence differences detected, particularly between the two final plastid genomes, we visually inspected select pairwise alignments in Geneious.

To visualize the genetic distances among selected assemblies and both individuals, we conducted principal coordinates analyses (PCoAs) using the uncapped dataset as well as the datasets capped at 2,000x and 500x as representatives for the complete range of different levels of sequencing coverage. In our plots, we centered the projections on the final plastid genome sequences (i.e., the assemblies generated with GetOrganelle for the read set capped at 500x), scaled the first two principal coordinates to a standard range from –1 to 1, and displayed the absolute variance (in bp) and the percentage of total variance along each axis within the plot. Since PCoAs can potentially distort pairwise distances between data points, we also plotted an overview of the genetic distances caused by changes in software (including seed selection) and coverage cap (including run replication). Moreover, we compared the pairwise genetic distances between Cb01A (set as origin) and Cb04B across software, levels of sequencing coverage, seed selection, and run replicate as a biologically meaningful standard for the assembly differences within each individual. All calculations and visualizations based on pairwise genetic distance matrices were conducted in R v.4.0.0 (R Development Core Team, 2019).

### 2.10. Visualizations of Region Length, Sequencing Coverage, and SNP Location

Three types of visualization were employed to illustrate the structural and sequence differences between select plastid genome assemblies of *C. bakuense*. First, we illustrated the length differences in the LSC, the SSC, and the two IRs across assemblies through an alignment overview of the four plastid genome regions using Geneious. Second, we visualized the depth of sequencing coverage across the entire plastid genome and in relation to the four genome regions and the position of its genes with PACVr v.1.0 using a calculation window of 250 bp (Gruenstaeudl and Jenke, 2020). Third, we determined and visualized the location of SNPs between assemblies and in relation to changes in sequencing coverage through pairwise comparisons of each assembly to the final genome sequence using MAFFT for sequence alignment and trimAl for SNP detection. We also visualized SNP locations relative to the four genome regions and the position of its genes using ShinyCircos v.29052020 (Yu et al., 2018). Visualizations were not produced for genome assemblies generated with GetOrganelle and NOVOPlasty under the 250x and 100x coverage cap levels, as these assemblies were identical to those generated under a coverage cap of 500x.

### 2.11. Phylogenetic Inference

To test if the sequence differences among the plastid genome assemblies of *C. bakuense* affect the phylogenetic placement of this species within *Calligonum*, we inferred the phylogenetic position of all plastid genome assemblies generated in this study among a taxonomically representative set of *Calligonum* species. Specifically, we retrieved 21 plastid genomes of *Calligonum* available from NCBI GenBank as of 30-Nov-2020 as well as the plastid genome of *Rheum palmatum* as an outgroup (GenBank accession KR816224; matching the study of Song et al., 2020) and combined these 22 genome records with the 28 genome assemblies generated here for the two individuals of *C. bakuense*. Then, we bioinformatically extracted 67 protein-coding regions, 17 introns, and 104 intergenic spacers from each of the 78 genome records using script 9 of Gruenstaeudl et al. (2018), automatically aligned the regions using MAFFT, and manually corrected the alignments where necessary. Extracting and aligning the different coding and non-coding regions individually (instead of conducting genome-wide alignments) reduces the probability of incorrect positional homology assessments during sequence alignment, especially if the input genomes differ in size (Gruenstaeudl et al., 2018). Even under these strict conditions, a total of 48 areas of unclear homology (mostly poly-A/T microsatellites; “hotspots” in Supplementary Table S1) were detected and removed from the alignments during manual alignment correction. The resulting alignments were concatenated to a combined matrix and their indels coded according to the simple indel coding scheme of Simmons and Ochoterena (2000) using 2matrix v.1.0 (Salinas and Little, 2014). A total of eight inversions (each less than 20 bp in length) were encountered within the alignments (Supplementary Table S1); to correctly include their phylogenetic information in our analyses, we coded them as presence-absence data, included this data alongside the regular indel information, and re-integrated their reverse-complemented sequences into the nucleotide alignments. The nucleotide matrix and the indel matrix were defined as separate partitions, and the best phylogenetic tree for this combined matrix was inferred under the maximum likelihood (ML) criterion using RAxML v.8.2.9 (Stamatakis, 2014). Clade support was inferred during tree inference through 1,000 bootstrap (BS) replicates generated with the rapid BS algorithm. To infer a phylogenetic position for *C. bakuense* within the genus *Calligonum*, we also conducted a second phylogenetic reconstruction involving only the two final plastid genome sequences of *C. bakuense*, the 21 genome records of *Calligonum* from NCBI GenBank, and the plastid genome of *Rheum palmatum* as an outgroup. For this second reconstruction, the best ML tree (including clade support) was inferred using RAxML as described above.

## 3. Results

### 3.1. Number of Sequence Reads

Genome skimming of the two individuals of *C. bakuense* resulted in a total of 151,567,745 paired raw sequence reads for Cb01A and a total of 166,362,653 paired raw sequence reads for Cb04B. Upon extraction of the plastid genome reads, we counted 5,062,912 paired reads (3.3% of raw reads) for Cb01A and 2,998,391 paired reads (1.8%) for Cb04B. Upon capping sequencing coverage, the read sets of Cb01A comprised 1,181,510 paired reads (0.78% of raw reads) under a level of sequencing coverage of 2,000x, 590,217 paired reads (0.39%) under 1,000x, 332,662 paired reads (0.22%) under 500x, 145,294 paired reads (0.10%) under 250x, 57,034 paired reads (0.04%) under 100x, and 27,913 paired reads (0.02%) under 50x. Similarly, the read sets of Cb04B comprised 1,149,375 paired reads (0.69% of raw reads) under a coverage cap of 2,000x, 571,982 paired reads (0.34%) under 1,000x, 333,839 paired reads (0.20%) under 500x, 140,425 paired reads (0.08%) under 250x, 54,922 paired reads (0.03%) under 100x, and 26,767 paired reads (0.02%) under 50x.

### 3.2. Impact of Software Choice

The choice of assembly software had a considerable effect on the number and size of the generated assembly contigs, the contiguity of the assemblies, sequence equality of the inferred IRs, and the time required to conduct each assembly (Table 1). While some software tools assembled the complete plastid genome of *C. bakuense* as a single contig, others did not. GetOrganelle and IOGA represented the extremes among the tested software tools: GetOrganelle succeeded in assembling the complete plastid genome as a single contig under nearly all settings, whereas IOGA failed in this task under all settings. Even under the original sequencing depth, which is representative of low-coverage nuclear genome skimming or even small nuclear genome sequencing projects, GetOrganelle successfully assembled the complete plastid genome of *C. bakuense* into a single, circular contig for both individuals and run replicates, precluding the need for any manual post-processing of the contigs. Similarly, NOVOPlasty succeeded in assembling the complete plastid genome of *C. bakuense* as a single, circular contig under the original sequencing depth for both individuals, run replicates, and seed sequences. For Cb01A, however, the assemblies generated with NOVOPlasty exhibited considerable size variability and often exceeded the length of the final plastid genome sequence; moreover, the inferred IRs were not identical in one of the assemblies. FastPlast also succeeded in assembling the complete plastid genome of *C. bakuense* as a single, circular contig under the original sequencing depth. However, the contigs produced for both individuals and both replicate runs lagged or exceeded the length of the final plastid genome sequences due to incomplete or duplicated sections of the IRs, ranging from 201 to 143 kb in Cb01A and from 192 kb to 175 kb in Cb04B. The smaller than expected contig lacked a section of the IRa, whereas the larger than expected contigs exhibited a duplication of sections of the LSC adjacent to the IRs, necessitating manual post-processing of the contigs and affecting the calculation of NGA50. IOGA, by contrast, did not succeed in assembling the complete plastid genome of *C. bakuense* as a single, complete contig under any setting. For both individuals, it generated more than 20 separate contigs, which represented only sections of the complete genome. Hence, the IOGA contigs had to be manually concatenated for both individuals and run replicates to generate circular assemblies. Moreover, the contigs assembled by IOGA for Cb01A did not imply identical IRs in one run replicate, indicating additional assembly problems. Computation times differed strongly across software tools and—in the case of IOGA and FastPlast—across run replicates, but were similar across different seed sequences in NOVOPlasty. Under the original sequencing depth, GetOrganelle and NOVOPlasty were typically the fastest to generate assembly contigs, whereas FastPlast and IOGA often required a multiple of their computation time. In summary, the plastid genome assemblies generated for *C. bakuense* with GetOrganelle and NOVOPlasty under the original sequencing depth were more consistent and required less, if any, manual post-processing than the assemblies generated with FastPlast and IOGA. Hence, we disregarded the latter two software tools during the more detailed evaluation of the impact of sequencing coverage on plastid genome assembly (Table 2).

**Table 1:** Assembly statistics for the plastid genomes of the two individuals of *C. bakuense* under study regarding the impact of assembly software choice, run replication, and seed selection.

**Asmb**.	**Cov**.	**Repl**.	**NOVO seed**	**Contigs**	**Largest contig (bp)**	**NGA50 (bp)**	**LGA50**	**IR equal**.	**Comp. time (h, min.)**
**Cb01A**									
FaPl	orig.	repl1		1	200,694	118,168	1	No	05 h 20 min
FaPl	orig.	repl2		1	143,261	135,202	1	No	06 h 40 min
FaPl	2,000x			1	162,404	162,128	1	Yes	01 h 16 min
FaPl	500x			1	163,292	162,896	1	Yes	24 min
GetO	orig.	repl1		1	162,128	162,128	1	Yes	44 min
GetO	orig.	repl2		1	162,128	162,128	1	Yes	44 min
GetO	2000x			2	118,241	118,215	1	Yes	01 h 08 min
**GetO**	**500x**			**1**	**162,128**	**162,128**	**1**	**Yes**	**20 min**
IOGA	orig.	repl1		21	89,039	88,068	1	Yes	09 h 42 min
IOGA	orig.	repl2		21	89,039	88,068	1	No	06 h 43 min
IOGA	2,000x			83	129,550	118,520	1	No	07 h 50 min
IOGA	500x			51	91,976	89,718	1	No	02 h 22 min
NOVO	orig.	repl1	seed1	1	170,093	131,660	1	No	01 h 05 min
NOVO	orig.	repl2	seed1	1	170,099	170,099	1	Yes	57 min
NOVO	2,000x		seed1	1	162,128	162,128	1	Yes	23 min
NOVO	500x		seed1	1	162,128	162,128	1	Yes	07 min
NOVO	orig.	repl1	seed2	1	162,128	162,128	1	Yes	01 h 05 min
NOVO	orig.	repl2	seed2	1	170,106	170,106	1	Yes	01 h 00 min
NOVO	2,000x		seed2	1	162,128	162,128	1	Yes	23 min
NOVO	500x		seed2	1	162,128	162,128	1	Yes	07 min
**Cb04B**									
FaPl	orig.	repl1		1	175,272	175,272	1	Yes	09 h 24 min
FaPl	orig.	repl2		1	192,943	118,215	1	No	03 h 36 min
FaPl	2,000x			1	163,890	162,129	1	Yes	01 h 16 min
FaPl	500x			1	163,292	163,292	1	Yes	24 min
GetO	orig.	repl1		1	162,129	162,129	1	Yes	04 h 16 min
GetO	orig.	repl2		1	162,129	162,129	1	Yes	01 h 04 min
GetO	2,000x			2	118,238	118,215	1	Yes	01 h 28 min
**GetO**	**500x**			**1**	**162,129**	**162,129**	**1**	**Yes**	**20 min**
IOGA	orig.	repl1		54	90,241	88,240	1	Yes	11 h 14 min
IOGA	orig.	repl2		85	90,630	87,507	1	Yes	07 h 42 min
IOGA	2,000x			102	55,966	27,790	2	No	08 h 17 min
IOGA	500x			40	75,285	74,394	1	No	03 h 18 min
NOVO	orig.	repl1	seed1	1	162,129	162,129	1	Yes	01 h 23 min
NOVO	orig.	repl2	seed1	1	162,129	162,129	1	Yes	01 h 24 min
NOVO	2,000x		seed1	1	162,129	162,129	1	Yes	20 min
NOVO	500x		seed1	1	162,129	162,129	1	Yes	06 min
NOVO	orig.	repl1	seed2	1	162,129	162,129	1	Yes	01 h 00 min
NOVO	orig.	repl2	seed2	1	162,129	162,129	1	Yes	01 h 32 min
NOVO	2,000x		seed2	1	162,129	162,129	1	Yes	20 min
NOVO	500x		seed2	1	162,129	162,129	1	Yes	06 min

*The assemblies that represent the final genome sequences are highlighted in bold. The assembly software tools compared are abbreviated as “FaPl” (for FastPlast), “GetO” (for GetOrganelles), “IOGA,” and “NOVO” (for NOVOPlasty). Run replicates are abbreviated as “repl1” or “repl2,” the original sequencing depth as “orig.” Other abbreviations used: asmb., assembly; comp., computation; cov., coverage; equal., equality in sequence; repl., replicate*.

**Table 2:** Assembly statistics for the plastid genomes of the two individuals of *C. bakuense* under study regarding the impact of different levels of sequencing coverage.

**Asmb**.	**Cov**.	**Compl**.	**Contigs**	**Largest contig (bp)**	**NGA50 (bp)**	**LGA50**	**IR length (bp)**	**IR equal**.	**Comp. time (h, min.)**
**Cb01A**									
GetO	orig.	Yes	1	162,128	162,128	1	30,526	Yes	44 min
GetO	2,000x	No	2	118,241	118,215	1	30,526	Yes	01 h 08 min
GetO	1,000x	No	1	62,295	n.s.d.	-	n.a.	n.a.	06 min
**GetO**	**500x**	**Yes**	**1**	**162,128**	**162,128**	**1**	**30,526**	**Yes**	**20 min**
GetO	250x	Yes	1	162,128	162,128	1	30,526	Yes	02 min
GetO	100x	Yes	1	162,128	162,128	1	30,526	Yes	01 min
GetO	50x	No	2	118,241	118,220	1	28,610	Yes	01 min
NOVO	orig.	Yes	1	170,093	131,660	1	44,559	No	01 h 05 min
NOVO	2,000x	Yes	1	162,128	162,128	1	30,526	Yes	23 min
NOVO	1,000x	Yes	1	162,128	162,128	1	30,526	Yes	11 min
NOVO	500x	Yes	1	162,128	162,128	1	30,526	Yes	07 min
NOVO	250x	Yes	1	162,128	162,128	1	30,526	Yes	05 min
NOVO	100x	Yes	1	162,128	162,128	1	30,526	Yes	02 min
NOVO	50x	No	1	117,861	117,849	1	n.a.	n.a.	09 min
**Cb04B**									
GetO	orig.	Yes	1	162,129	162,129	1	30,526	Yes	04 h 16 min
GetO	2,000x	No	2	118,238	118,215	1	30,526	Yes	01 h 28 min
GetO	1000x	No	1	67,160	n.s.d.	-	n.a.	n.a.	06 min
**GetO**	**500x**	**Yes**	**1**	**162,129**	**162,129**	**1**	**30,526**	**Yes**	**20 min**
GetO	250x	Yes	1	162,129	162,129	1	30,526	Yes	02 min
GetO	100x	Yes	1	162,129	162,129	1	30,526	Yes	01 min
GetO	50x	No	2	118,236	118,215	1	30,526	Yes	01 min
NOVO	orig.	Yes	1	162,129	162,129	1	30,526	Yes	01 h 23 min
NOVO	2,000x	Yes	1	162,129	162,129	1	30,526	Yes	20 min
NOVO	1,000x	Yes	1	162,129	162,129	1	30,526	Yes	15 min
NOVO	500x	Yes	1	162,129	162,129	1	30,526	Yes	06 min
NOVO	250x	Yes	1	162,129	162,129	1	30,476	Yes	04 min
NOVO	100x	No	4	112,054	112,054	1	30,526	Yes	07 min
NOVO	50x	No	1	75,891	n.s.d.	-	n.a.	n.a.	24 min

*For assemblies under the original sequencing depth, only the first run replicate is displayed; for all assemblies performed with NOVOPlasty, seed sequence 1 was employed. Abbreviations used: compl., complete genome assembled; n.a., not applicable; n.s.d., no similarity detected by QUAST; all other abbreviations used as in Table 1. The assemblies that represent the final genome sequences are highlighted in bold*.

### 3.3. Impact of Sequencing Coverage

The sequencing coverage also had a considerable effect on the number and size of the generated assembly contigs, the contiguity of the assemblies, sequence equality of the inferred IRs, and the time required to conduct each assembly. We observed that GetOrganelle assembled the complete plastid genome of *C. bakuense* into the same circular contig under the original sequencing depth and all levels of sequencing coverage between and including 100x and 500x for both samples under study (Table 2). For sequencing coverage levels of 50x, 1,000x, and 2,000x, however, it generated two separate contigs that had to be concatenated to create a complete genome sequence. The breakpoint between these contigs was typically located at the junction site between IRb and the SSC, indicating that this non-contiguity was correlated with the quadripartite genome structure. NOVOPlasty seemed insensitive to changes in sequencing coverage across medium depth ranges, as it assembled the same circular complete plastid genome sequence under all levels between and including 500x and 2,000x for both samples (Table 2). For sequencing coverage above and below that range, however, NOVOPlasty was unable to assemble the same contig and instead generated either multiple smaller contigs, incomplete contigs, or contigs with unequal IR size. The single circular contig generated by GetOrganelle under sequence coverages of 100x–500x and by NOVOPlasty under 500x or 2,000x was identical within each individual, and thus identical to the designated final plastid genomes of the *C. bakuense* individuals (i.e.,GetOrganelle under a sequencing coverage of 500x). Hence, at a sequencing coverage of 500x, both GetOrganelle and NOVOPlasty immediately and repeatably produced a complete plastid genome assembly for both individuals.

A strong variability in contig number, contig sequence, and contig length with regard to sequencing coverage was detected for assemblies generated with FastPlast and IOGA (Table 1). All genome assemblies generated by FastPlast under different levels of sequencing coverage exhibited different contig lengths. Moreover, the IRs of the assembled plastid genomes were found to be identical within assemblies only under the capped read sets as well as replicate run 1 of the uncapped read set in Cb04B. The assembly process of IOGA appeared to be even more sensitive to changes in sequencing coverage: for individual Cb01A, IOGA assembled 21 contigs under the original read set, 83 contigs under a coverage cap of 2,000x, and 51 contigs under a coverage cap of 500x; for Cb04B, the software generated between 54 and 85 contigs under the original read set (depending on the run replicate), 102 contigs under a coverage cap of 2,000x, and 40 contigs under a coverage cap of 500x. While at least half of the final genome sequence was encompassed within a single contig in all but one of these cases, the assembly results for each level of sequencing coverage had to be manually concatenated to generate complete plastid genomes. In addition to this high sensitivity to sequencing coverage, differences between replicate runs also indicated low reproducibility for sequence assemblies by both FastPlast and IOGA.

Computation time differed strongly across different assembly software and sequencing coverage and was generally correlated with the size of the input dataset: datasets with a capped sequencing coverage were typically analyzed faster than the original datasets (Tables 1, 2). For a sequencing coverage of 500x, NOVOPlasty was the software that achieved a complete plastid genome assembly for *C. bakuense* in the shortest amount of time (7 min. and 6 min. for Cb01A and Cb04B, respectively); for lower levels of sequencing coverage, GetOrganelle was the software to achieve complete assemblies fastest.

**Figure 2:**
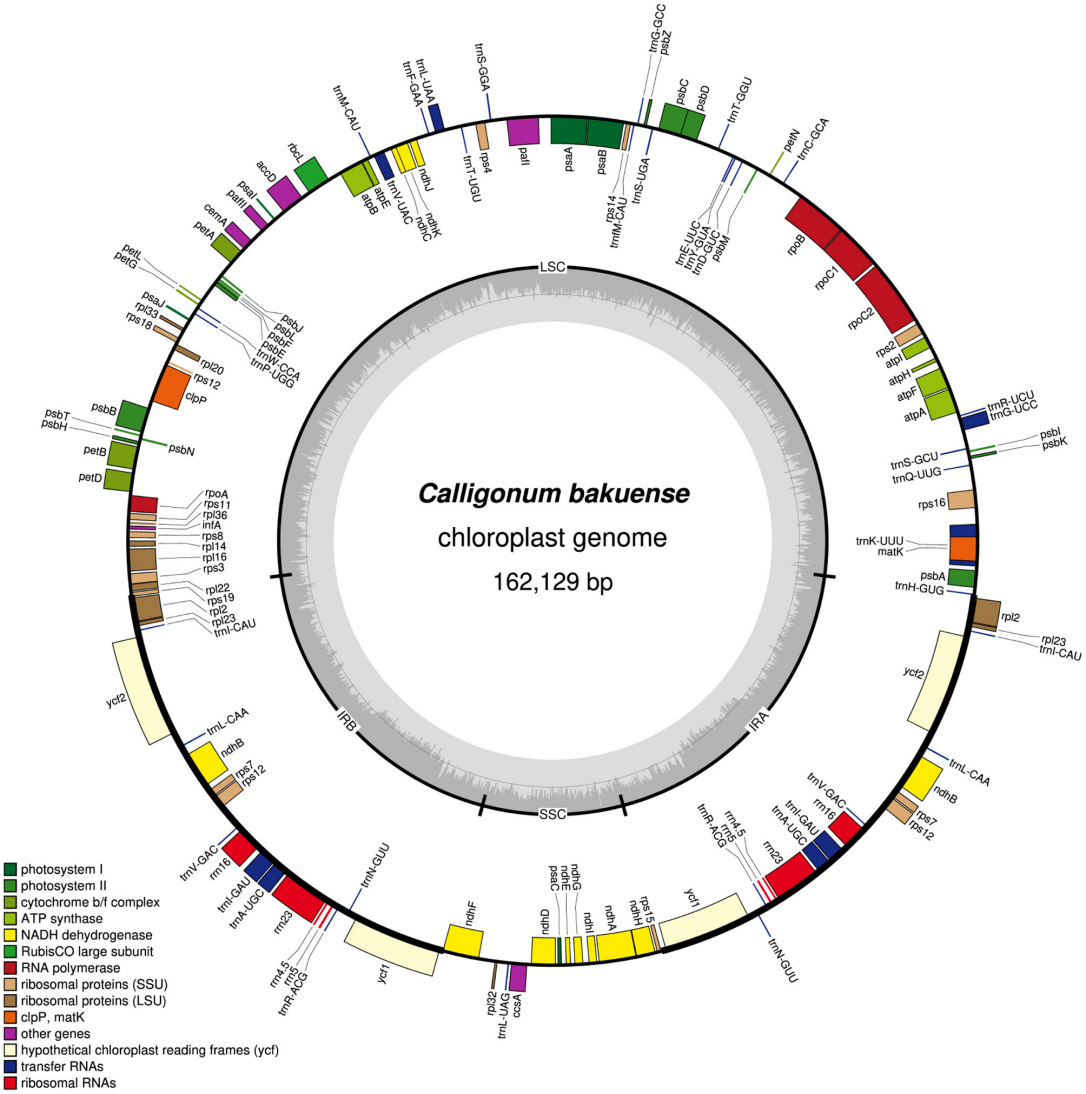
Map of the complete plastid genome of individual Cb01A of *C. bakuense* as assembled by GetOrganelle under a coverage cap of 500x. This assembly represents the final plastid genome sequence for Cb01A.

In summary, we found that among the four assembly software tools tested, GetOrganelle and NOVOPlasty usually generated plastid genome assemblies that were identical in both length and sequence across run replicates and most levels of sequencing coverage. Occasional occurrences of more than two contigs generated per assembly run (e.g., GetOrganelle under a sequencing coverage of 2,000x) do not invalidate this observation, as the break point between such contigs was typically located at the junction between IRb and the SSC, which is a natural break point in a circular quadripartite genome. Overall, GetOrganelle slightly outperformed NOVOPlasty: it produced the full plastid genome in one contig already at lower sequencing coverage and had higher assembly accuracy, as some assembly results generated by NOVOPlasty contained sequence replications that extended the plastid genome sequence beyond its actual size (e.g., assembly of Cb01A under the original sequencing depth). We, therefore, considered the plastid genome sequences generated with GetOrganelle for the two individuals of *C. bakuense* as the best results and submitted them as official plastid genome sequences for the species to GenBank (accessions MT806099 for Cb01A and MT806098 for Cb04B; Figure 2). Based on these sequences, the plastid genomes of Cb01A and Cb04B are almost identical and differ only by three nucleotides: a missing adenine in the intergenic spacer between the genes *ndhF* and *rpl32* in Cb01A, an additional thymine within a poly-T-microsatellite in the spacer between *rps16* and *trnQ-UUG* in Cb04B, and an additional thymine within a poly-T microsatellite in the spacer between *pafI* and *trnS-GGA* in Cb01A. Plastid genome diversity within *C. bakuense* is, thus, extremely low, but not zero.

### 3.4. Characterization of Assembly Differences

**Figure 3:**
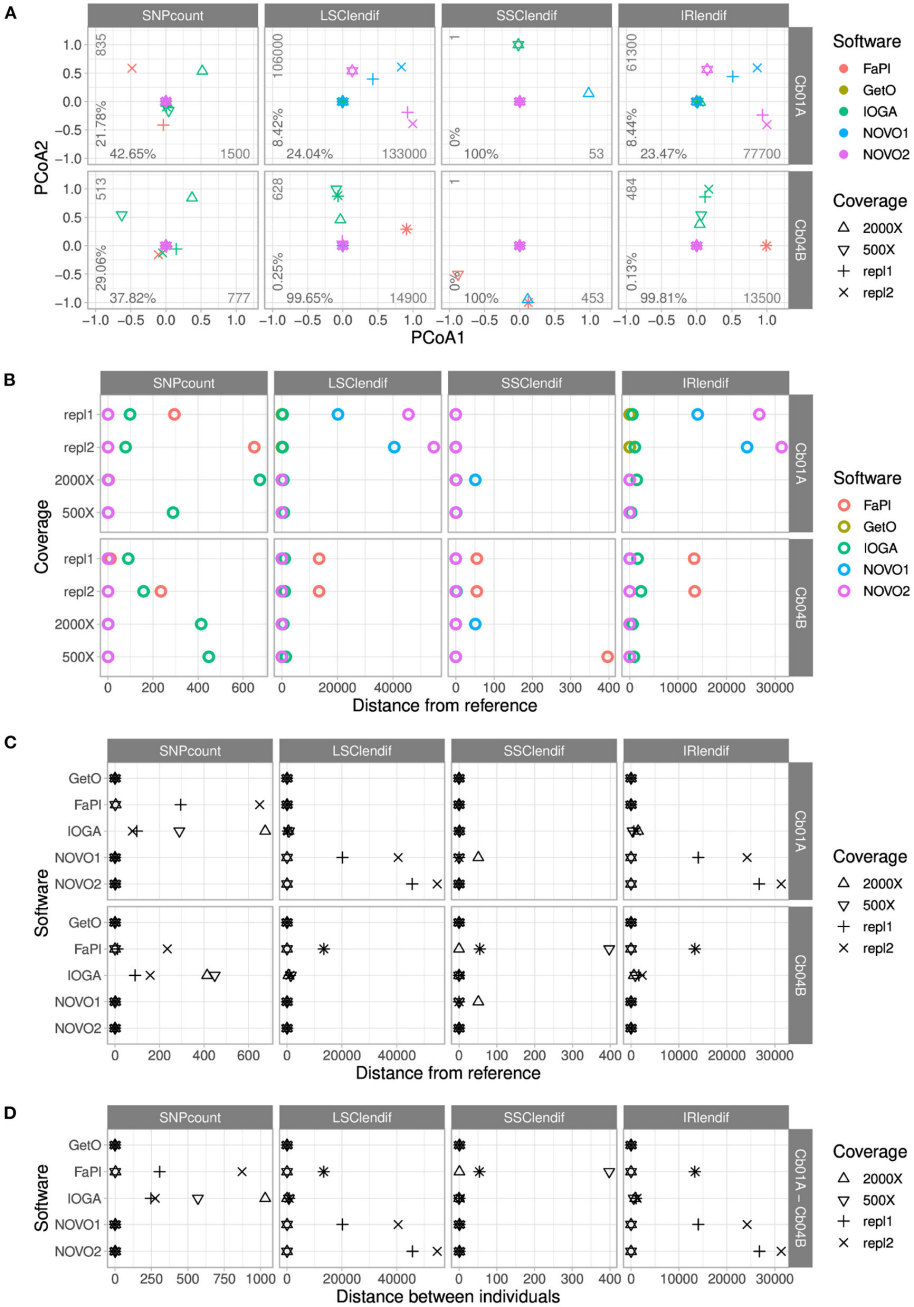
Comparisons of the number of SNPs and the lengths of the four genome regions across the plastid genome assemblies of *C. bakuense* as generated by different assembly software and levels of sequencing coverage. Subplot **(A)** displays the results of PCoAs, subplots **(B,C)** the results of comparisons between a target assembly and the final plastid genome sequence, and subplot **(D)** the results of assembly comparisons between the two individuals of *C. bakuense* under study. In the PCoA plots, the percentages indicate the variance explained by the first (x-axis) and second (y-axis) principal coordinate, and the integers express the range of the data. The abbreviations for the four distance metrics are: “SNPcount” for the total number of SNPs between two assemblies; “LSClendif,” “SSClendif,” and “IRlendif” for the differences in sequence length in the LSC, SSC, and IR between two assemblies, respectively.

PCoA of the number of SNPs and the length of each of the four plastid genome regions indicated the presence of a complex pattern of differences among plastid genome assemblies of different software tools and levels of sequencing coverage (Figure 3A). Assemblies produced by different software tools were heterogeneous in both length and sequence for both individuals and differed by additional SNPs and the length of one or more plastid genome regions. In Cb04B, the first coordinate of the PCoA explained nearly the entire variance in the lengths of the four plastid genome regions, indicating the presence of one extreme or two nearly identical outlier assemblies. In Cb01A, the first coordinate of the PCoA similarly explained nearly the entire, comparatively low variance for the SSC length, but not for the lengths of the LSC and the IRs, where more diversity among a greater number of outliers was identified. For the number of SNPs, the first two PCoA coordinates together explained >60% of the variance in both individuals, although overall variance for Cb01A was greater than for Cb04B according to the absolute variance values.

The comparison of pairwise genetic distances between the assemblies of different software tools highlighted the presence of SNPs between the final genome sequences and the assemblies generated with FastPlast and IOGA (Figure 3B). This contrasts with the presence of IR and LSC length differences between the final genome sequences and the assemblies generated with NOVOPlasty (especially in Cb01A) and FastPlast (especially in Cb04B). The overall similarity of the length difference patterns for the LSC and the IR suggests that length deviations in either region are often compensated by a corresponding change in the other region during genome assembly, rather than changes of the SSC.

The comparison of pairwise genetic distances between the assemblies of different levels of sequencing coverage highlighted that the observed length and sequence deviations from the final genome sequences were not constant across different levels (Figure 3C); only the assemblies generated with GetOrganelle were found to be unaffected by alterations in sequencing coverage. For assemblies generated with IOGA, for example, the reduction of sequencing coverage had a complex but strong effect on SNP count and region length, as it correlated with a decrease of the number of SNPs and the IR/LSC length difference in Cb01A but an increase of both factors in Cb04B. A similar pattern was found for assemblies generated with FastPlast and, for Cb01A, also for NOVOPlasty. GetOrganelle was the only assembly software found to produce assemblies with the same sequence and region lengths across all evaluated assembly parameters.

The comparison of genetic distances between the assemblies of the two individuals of *C. bakuense* demonstrated that only GetOrganelle consistently and repeatedly generated the final plastid genome sequence for each individual under study (Figure 3D). We did not find any SNPs between the assemblies produced by GetOrganelle for the two individuals except for two nucleotide differences in the LSC (which were neutral regarding the overall length difference due to their occurrence in different individuals) and one in the SSC. Under FastPlast and IOGA, by contrast, the number of SNPs detected between the two assemblies was much greater and even exceeded the threshold of 1,000 nucleotide differences in the case of IOGA. Moreover, under both FastPlast and IOGA the number of SNPs between different assemblies of the same individual did not sum up to the number of SNPs between individuals, suggesting that at least some of the SNPs were shared between the assemblies of the same individual. The differences in LSC and IR length for assemblies generated with NOVOPlasty appeared to be correlated, suggesting that a length deviation in one region was compensated for by a corresponding change in the other region rather than a change in SSC length. Furthermore, visual examination of the assemblies indicated that several assemblies generated with IOGA under higher levels of sequencing coverage deviated from the other assemblies by insertions ranging from 170 and 334 bp; these insertions often had little, if any, similarity to other regions of the plastid genome.

**Figure 4:**
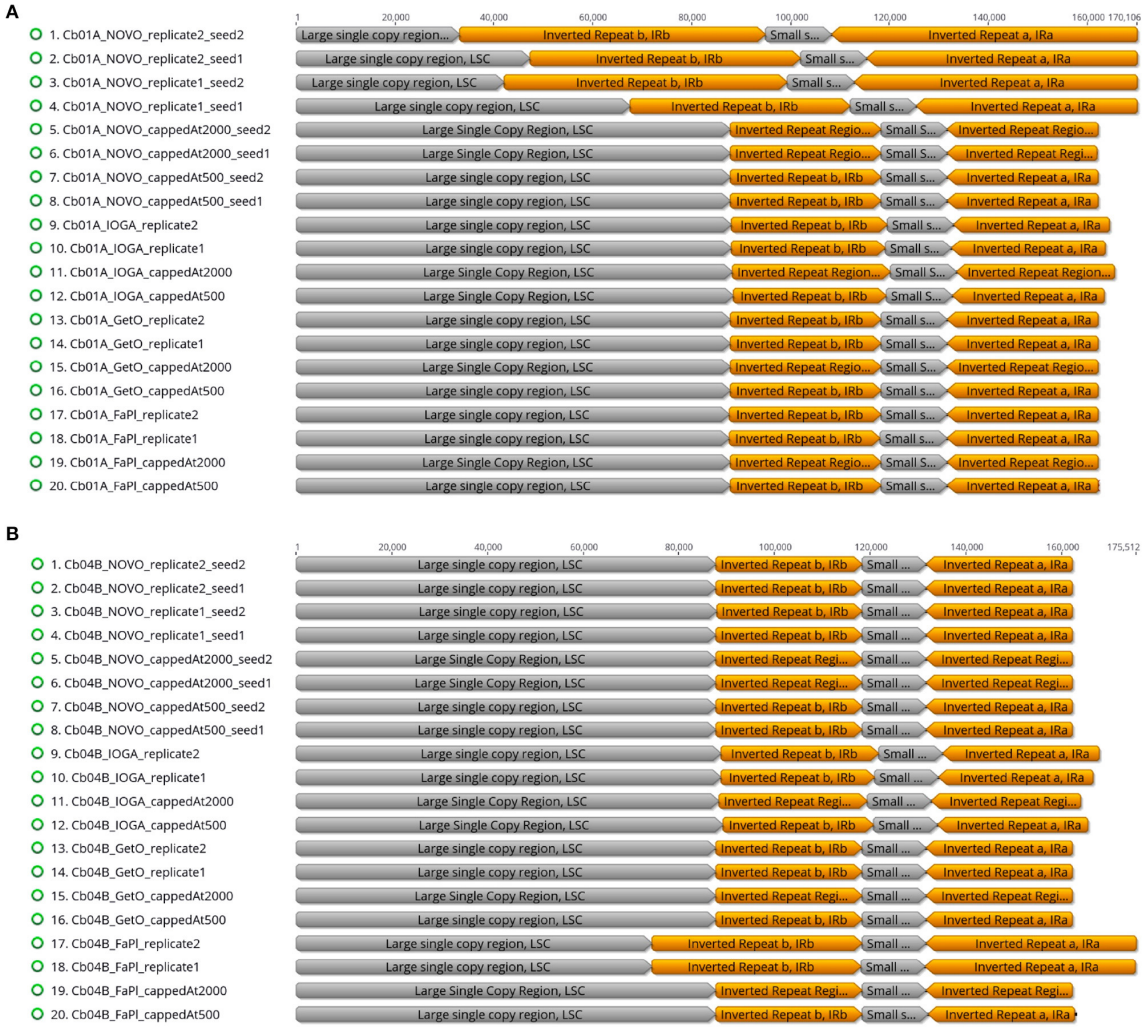
Overview of the relative lengths of the LSC, the SSC, and the two IRs across the plastid genome assemblies of the individuals Cb01A **(A)** and Cb04B **(B)** of *C. bakuense* as generated by different assembly software and levels of sequencing coverage.

The visual comparison of the lengths of the four plastid genome regions across different genome assemblies indicated that the differences in total genome length were primarily correlated with length changes in the LSC and the IRs (Figure 4). While the length of the SSC was virtually constant across all software tools and sequencing coverage (~13,400 bp; Supplementary Table S2), the length of the IR was highly sensitive to the precise assembly conditions. Especially in assemblies generated with NOVOPlasty for Cb01A as well as with FastPlast for Cb04B, the IR lengths varied by a factor of 1.5 to 2, which was partially compensated for by a corresponding reduction of the LSC length, sometimes to less than half of the length displayed in other assemblies. A complete list of the lengths of the four plastid genome regions in relation to the different software tools, levels of sequencing coverage, seed sequences, and run replicates is given in Supplementary Table S2 for Cb01A and Supplementary Table S3 for Cb04B (Supplementary Material).

### 3.5. Differences in Gene Content and Annotations

The nucleotide and length differences between the assembled plastid genomes were located in both the coding and the non-coding sections of the genomes and often manifested themselves as differences in gene content (Table 3). Specifically, the annotated sequences of several assemblies either lacked certain protein- and tRNA-coding genes due to missing genome sections or exhibited non-functional protein-coding genes due to internal stop codons caused by nucleotide polymorphisms. All assemblies generated with IOGA, for example, exhibited housekeeping genes with internal stop codons, which are indicative of an incorrect assembly. Among the assemblies generated with FastPlast, replicate runs 1 and 2 for Cb01A and replicate run 2 for Cb04B under the original sequencing depth as well as the assembly of Cb04B under a coverage cap of 500x produced gene sequences with internal stop codons. Similarly, the length differences between the four plastid genome regions across the assemblies generated with NOVOPlasty for Cb01A correlated with a lack of up to 17 different genes compared to the final genome sequence of that plant individual, even when all assembly contigs were concatenated to a super-contig; this result was observed for both seed sequences and, thus, appears to be independent of the internal start point of the genome assembly. All of the missing genome regions in the assemblies generated with NOVOPlasty were noticeably located at the 5' end of the LSC, suggesting a potential bias in the assembly of this genome region. All plastid genome assemblies generated with GetOrganelle, by contrast, exhibited a complete gene complement and the full genome size.

**Table 3:** Overview of incorrect or missing annotations among the plastid genome assemblies of *C. bakuense* as generated under different assembly software, sequencing coverage, seed sequences, and run replicates.

**Asmb**.	**Cov**.	**Repl**.	**NOVO seed**	**Internal stop codons in translation**	**No DNA sequence at this position**
**Cb01A**					
FaPl	orig.	repl1		rpl23^a,b^, rrn16^a,b^	
FaPl	orig.	repl2		rpl2^a,b^, ycf2^a,b^, rpl23^a^	
FaPl	2,000x				
FaPl	500x				
IOGA	orig.	repl1		psbA, rpl23^a,b^, ycf2^a,b^, rrn16^a,b^	
IOGA	orig.	repl2		psbA, ycf2^a,b^	
IOGA	2,000x			psbA, ycf2^a,b^, ycf1^a,b^, ndhH	
IOGA	500x			psbA, rps2^a,b^, ycf2^a,b^, ndhH	trnH-GUG
NOVO	orig.	repl1	seed1		trnH-GUG, psbA, trnK-UUU, matK, rps16
NOVO	orig.	repl2	seed1		trnH-GUG, psbA, trnK-UUU, matK, rps16, trnQ-UUG, psbK, psbI, trnS-GCU, trnG-UCC, trnR-UCU, atpA, atpF, atpH, atpI, rps2, rpoC2
NOVO	2,000x		seed1		
NOVO	500x		seed1		
NOVO	orig.	repl1	seed2		trnH-GUG, psbA, trnK-UUU, matK, rps16, trnQ-UUG, psbK, psbI, trnS-GCU, trnG-UCC, trnR-UCU, atpA, atpF, atpH, atpI, rps2, rpoC2
NOVO	orig.	repl2	seed2		trnH-GUG, psbA, trnK-UUU, matK, rps16, trnQ-UUG, psbK, psbI, trnS-GCU, trnG-UCC, trnR-UCU, atpA, atpF, atpH, atpI, rps2, rpoC2
NOVO	2,000x		seed2		
NOVO	500x		seed2		
**Cb04B**					
FaPl	orig.	repl1			
FaPl	orig.	repl2		rpl23^a^	
FaPl	2,000x				
FaPl	500x			ndhF	
IOGA	orig.	repl1		psbA, rpl23^b^, rpl2^a^	
IOGA	orig.	repl2		psbA, ndhH, rpl23^a^, rpl2^a,b^	
IOGA	2,000x			psbA, petB	
IOGA	500x			psbA, rps23^a,b^	

*All plastid genome assemblies generated with GetOrganelle for both individuals and with NOVOPlasty for Cb04B exhibited a complete gene complement and a full genome size and are, thus, not listed. The last column denotes cases of incomplete genomes despite the assembly being circular and indicated as complete by the assembly software. A location in IRa is indicated^a^, a location in IRb^b^. Abbreviations used as in Table 1*.

### 3.6. Sequencing Coverage and SNP Location

**Figure 5:**
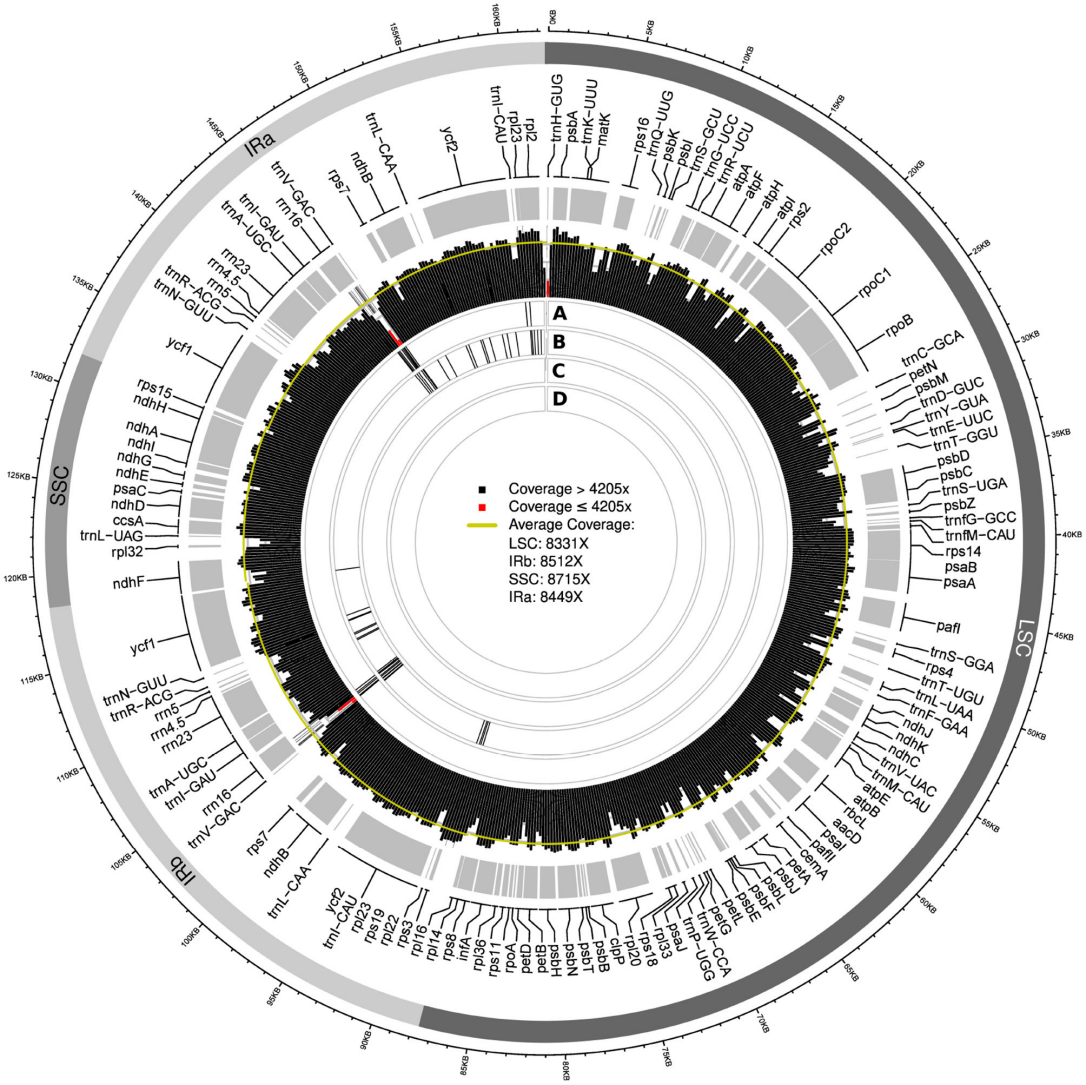
Visualization of the sequencing coverage across the plastid genome of individual Cb01A as generated with FastPlast and the location of SNPs of assemblies generated under different levels of sequencing coverage. Red bars in the visualization of sequencing coverage indicate calculation windows with a depth equal to, or less than, 50% of genome-wide sequencing depth. The four rings beneath the coverage visualization indicate the location of SNPs relative to the final genome sequence for the following assemblies: replicate run 1 (A) and 2 (B) under the original sequencing depth; a coverage cap of 2,000x (C); a coverage cap of 500x (D). Black bars within each ring represent the occurrence of three SNPs per 100 bp.

Visualizing the location of SNPs across the plastid genome assemblies indicated a possible association of their location with regions of low sequencing coverage. The genome-wide sequencing depth based on the uncapped datasets was 8,410x for the final plastid genome of Cb01A and 5,430x for that of Cb04B. Among the assemblies generated with different assembly software, a considerable number exhibited SNPs when compared to the final genome sequence. Notably, these SNPs were often associated with regions of reduced sequencing coverage. For example, the IRs of the plastid genome assemblies of Cb01A generated with FastPlast contained two adjacent calculation windows with a sequencing coverage of 1,200x and 2,300x, respectively; these depths represent only 14% and 27% of the genome-wide sequencing depth (Figure 5). The two windows were located between the tRNA genes *trnV-GAC* and *trnI-GAU* and covered parts of the gene coding for the 16S rRNA subunit (*rrn16*). Compared to the final genome sequence of Cb01A, the assemblies of both replicate runs exhibited a high density of SNPs in the very same region (Figure 5, circles A and B); SNPs outside this particular region also existed but were clustered less densely, if at all. Similarly, a high density of SNPs was found in replicate run 2 at the replication origin of the genome, which also exhibits a considerably reduced sequencing coverage (Figure 5, circle B); however, the reduced sequencing coverage at the replication origin represents an artifact introduced by the mapping software during the extraction of plastid genome reads from the raw read set and should, thus, not be seen as a region with naturally reduced sequencing coverage. The assemblies generated with FastPlast under the capped read sets, by contrast, did not exhibit SNPs compared to the final genome sequence (Figure 5, circles C and D). A similar interdependence between the location of SNPs and regions with reduced sequencing coverage was observed for the assemblies of Cb01A generated with IOGA (Supplementary Figure S1); the amount and the distribution of SNPs in comparison to the final genome sequence were, however, greater than in the assemblies with FastPlast and neither restricted to the IRs nor any particular read set. By comparison, the plastid genome assemblies generated with GetOrganelle or NOVOPlasty did not display any SNPs in comparison to the final genome sequence, irrespective of a cap on sequencing coverage.

### 3.7. Phylogenetic Inference

The results of our phylogenetic tree reconstructions on the combined set of all plastid genome assemblies of *C. bakuense* plus the 21 plastid genome records of other species of *Calligonum* and the outgroup did not indicate that the sequence variability across the assemblies generated in this study was large enough to affect the phylogenetic placement of *C. bakuense* within *Calligonum* (Supplementary Figures S2, S3). While the different genome assemblies of *C. bakuense* did not cluster by assembly software or level of sequencing coverage, they did exhibit a noticeable clustering by plant individual. Specifically, a strong clustering by plant individual was observed when sequence insertions and deletions (indels) of the underlying matrix were coded and included in the phylogenetic reconstruction (Supplementary Figure S3), whereas no such clustering was observed without the coding of indels (Supplementary Figure S2). Moreover, we found that the nucleotide differences between the majority of our assemblies were not or only minimally phylogenetically informative and, thus, did not result in the identification of specific clades among the assembly sequences. The observed sequence differences among the assemblies may nonetheless be large enough to influence intra-specific evolutionary analyses of *C. bakuense*.

**Figure 6:**
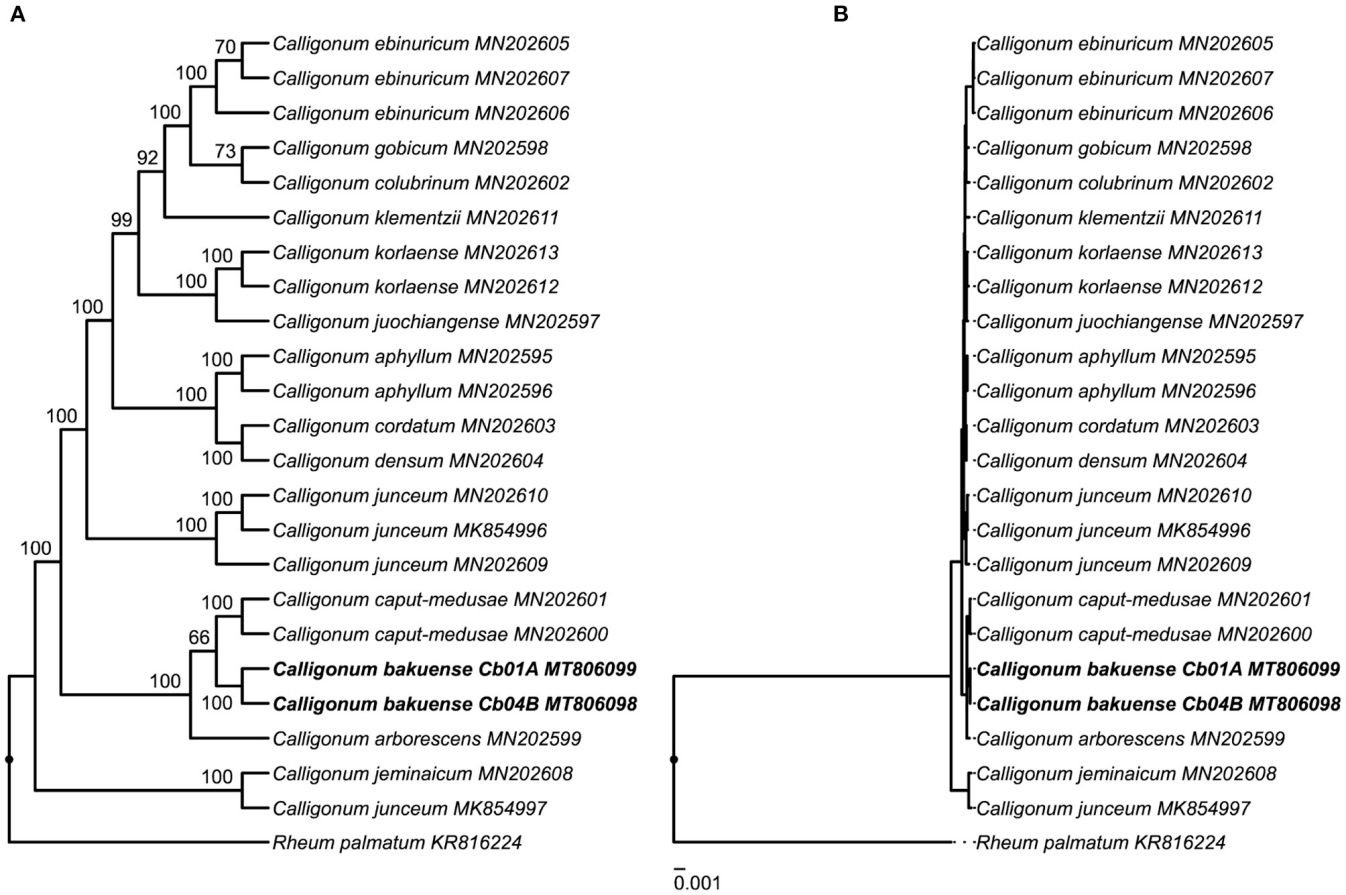
Phylogenetic position of *C. bakuense* among other species of *Calligonum*. *C. bakuense* is represented by the final plastid genomes of individuals Cb01A and Cb04B, which are highlighted in bold. The displayed phylogenetic tree represents the best tree inferred under ML, visualized as **(A)** cladogram with bootstrap node support (given above branches) and **(B)** the corresponding phylogram with exact branch lengths.

The results of our phylogenetic tree reconstruction to infer the phylogenetic position of *C. bakuense* among other species of *Calligonum* recovered the final plastid genomes of *C. bakuense* as sister to *C. caput-medusae* (Figure 6 and Supplementary Figure S2). The sister relationship between *C. bakuense* and *C. caput-medusae* was weakly supported (BS 66%) but both taxa were recovered as part of a fully-supported clade alongside *C. arborescens*. Overall, the reconstruction recovered the same phylogenetic relationships as reported by Song et al. (2020), indicating that the inclusion of *C. bakuense* did not alter the tree reconstruction of the genus.

## 4. Discussion

### 4.1. Phylogenetic Position of *C. bakuense* Based on Complete Plastid Genomes

This investigation is the first to report the complete plastid genome of *C. bakuense* and, thus, advances our understanding of *Calligonum*, as knowledge of the plastid genome of this Caucasian endemic supports research on the evolutionary diversification of the genus. For example, our analyses underscore the potential of complete plastid genome sequences for resolving species-level relationships in *Calligonum* (e.g., Song et al., 2020), whereas individual regions of the plastid genome appear to yield insufficient phylogenetic information (e.g., Tavakkoli et al., 2010). The results of our phylogenetic analyses (Figure 6) only partially agree with the current taxonomic classification of *Calligonum*. *Calligonum bakuense* is considered a member of sect. *Calligonum*, yet was recovered as part of a clade formed by individuals of *C. arborescens* and *C. caput-medusae*, both of which are members of sect. *Medusa* SOSK. ET ALEXANDR (Soskov, 2011). The current sectional classification of *Calligonum* is primarily based on differences in fruit morphology and probably not natural, as suggested by Song et al. (2020); our results provide further evidence for this interpretation. The phylogenetic position of *C. bakuense* in a clade with *C. arborescens* and *C. caput-medusae* may indicate that *C. bakuense* represents an isolated lineage endemic to Azerbaijan in a Caucasian-central Asian clade. *Calligonum bakuense* occurs on the west coast of the Caspian Sea, whereas *C. arborescens* and *C. caput-medusae* both grow in steppe habitats east of the Caspian Sea, ranging from Turkmenistan to China.

Due to similarities in fruit morphology and its tetraploid nature (2n = 36; Bolkhovskikh et al., 1969), *C. bakuense* was hypothesized to be an allotetraploid that arose from ancestors of *C. polygonoides* L. and *C. acanthopterum* I.G. BORSHCH (Soskov and Akhmed-Zade, 1974). While *C. polygonoides* is widespread and also occurs in Azerbaijan (Karjagin, 1952), *C. acanthopterum* is known only from Kazakhstan and Turkmenistan. By contrast, the widespread species *C. aphyllum*, which is distributed from North Africa to the Caucasus (including Azerbaijan) and China, is morphologically distinct from *C. bakuense* (e.g., winged fruits that lack bristles) and probably not a close relative to *C. bakuense*. Future phylogenetic investigations should, thus, increase both the taxon sampling and, where possible, the geographic representation of the more widespread taxa of *Calligonum* such as *C. polygonoides*. Since the relationships among *C. bakuense, C. arborescens*, and *C. caput-medusae* were unsupported when only the coding sections of the plastid genome were used for phylogenetic reconstruction (trees not shown), our results corroborate the observation that the inclusion of the non-coding sections of the plastid genome (i.e., introns and intergenic spacers) in a genus-wide plastid phylogenomic analysis represents an important aspect in clarifying the phylogenetic history of angiosperm genera with low genetic distances among species (e.g., *Gynoxys*; Escobari et al., 2021). The inclusion of phylogenetic information from the nuclear genome in future investigations will likely assist in clarifying possible reticulate speciation events within *Calligonum*.

The three nucleotide differences detected between the plastid genomes of the two individuals of *C. bakuense* are comparatively few but could be in the same range as those of other narrow endemic plant species. While intra-specific comparisons of complete plastid genomes are still rare (e.g., Jiang et al., 2017; Teshome et al., 2020), published studies of endemics often report only a handful of SNPs between plant individuals. The narrow endemic *Pinus torreyana*, for example, had five SNPs between the plastid genomes of two individuals from both parts of its disjunct distribution range (Whittall et al., 2010, indels not reported). Similarly, at least two SNPs and one indel were found between two plastid genomes of *Fagus multinervis*, which is endemic to Ulleung Island near the South Korean coast (Yang et al., 2020). While plastid genome sequences of *C. bakuense* provide valuable background on the evolutionary history of this species, further analysis of its nuclear genomic diversity remains necessary for a sound conservation genetic assessment.

### 4.2. Impact of Software Choice on Plastid Genome Assembly

By comparing the assembly contigs of *C. bakuense* that were generated with four different software tools, we found that assembly software choice can have an inordinate influence on the inferred plastid genome sequences and that the results of some tools need to be treated with caution. Among the differences across the assemblies were the presence of SNPs and indels (compared to the final genome sequences), the incorrect absence of entire genes or loss of their functionality, and the expansion and contraction of the IRs (as well as compensatory length changes in the LSC). Such occurrences have been occasionally interpreted in an evolutionary context (e.g., Mohanta et al., 2020), but it stands to reason that at least some of the differences between the plastid genomes of closely related species may have a more technical origin, as recently demonstrated by Freudenthal et al. (2020). The results of this investigation support the hypothesis that differences among plastid genome assemblies may also be technical in nature. We found that the plastid genome assemblies of different software tools exhibited considerably different genome sequences despite employing the same input data and that some of the assembly contigs could not be replicated in different runs of the same software (Table 1). Only the software GetOrganelle was found to generate consistent and repeatable results for both datasets. The software FastPlast, by contrast, was found to be prone to the introduction of SNPs and, in some cases, also structural deviation among the assemblies. NOVOPlasty introduced few, if any, SNPs compared to the final genome sequences but exhibited a tendency for generating structural deviations, which even occurred when the same assembly was conducted with different seed sequences. The assemblies generated with IOGA were fragmentary in all cases and exhibited numerous SNPs and structural deviations compared to the final genome sequences. Worse still, we found that many of these sequence deviations generated by IOGA would result in incorrect conclusions about gene content and functionality when compared to the final genome sequences (Table 3). We, therefore, concur with Freudenthal et al. (2020) that users should abstain from employing the software IOGA (which is no longer maintained) for plastid genome assembly and that the assemblers FastPlast and NOVOPlasty should be employed with caution. We also concur with the suggestion that the replication of assembly results across different software runs and seed sequences (where applicable) are beneficial precautions in the generation of trustworthy plastid genome sequences.

Our results do not imply that the assemblies generated with GetOrganelle necessarily represent true plastid genome sequences for *C. bakuense*. It is possible for a software tool to consistently and repeatably produce incorrect results, and we also cannot rule out the presence of more than one unique plastid genome per plant individual (Scarcelli et al., 2016; Wang and Lanfear, 2019). However, the software tools FastPlast and NOVOPlasty produced the same genome sequence as identified through GetOrganelle under some of the evaluated settings. We, therefore, considered the plastid genome assemblies generated with GetOrganelle under the read sets capped at a sequencing coverage of 500x as the most likely genome sequences for the two individuals of *C. bakuense* and employed them as the final plastid genomes. Aside from the idiosyncrasies introduced by different assembly software, the observed differences among the plastid genome assemblies may also be the result of nucleotide polymorphism among the input reads (Scarcelli et al., 2016). Such polymorphism within the read set could represent genuinely different variants of the plastid genome (i.e., heteroplasmy; Walker et al., 2015; Wang and Lanfear, 2019), genomic transfers of sections of the plastid to the nuclear or the mitochondrial genome, followed by a pseudogenization of the transferred regions (Ruhlman and Jansen, 2014), or sequencing errors during data generation (Nakamura et al., 2011), and may be decoded differently by different assembly software.

### 4.3. Impact of Sequencing Coverage on Plastid Genome Assembly

By comparing the assembly contigs of *C. bakuense* generated under different levels of sequencing coverage, we found that sequencing coverage can also have an impact on plastid genome assembly. Specifically, we found that the capping of sequencing coverage prior to genome assembly had a measurable effect on the number of assembly contigs constructed, the nucleotide sequences of these contigs, the length of the different plastid genome regions (particularly the IRs), and the number of valid gene annotations. The effects of capping sequencing coverage were measurable in both samples and suggested the trend that a sequencing depth between 100x and 500x rendered the assemblies relatively consistent in sequence and length (Table 2). Specifically, a sequencing depth between 100x and 500x appeared to ensure replicability of the genome assemblies with GetOrganelle and NOVOPlasty, whereas levels of sequencing coverage above and below that range did not enable a complete plastid genome assembly. A similar albeit slightly lower range of optimal sequencing depth for the assembly of plastid genomes has been reported for PacBio sequencing data (i.e., 50–200x; Soorni et al., 2017) and is in line with observations on the absolute minimum sequencing coverage for the reliable plastid genome assembly (i.e., 30–50x; Twyford and Ness, 2017; Sharpe et al., 2020). In practice, an amount of approximately two to 10 million Illumina read pairs of 150 bp length per read, generated from DNA fragments with an average length of 300 bp, can cover a plastid genome of approximately 160,000 bp with a sequencing coverage of 100x to 500x. This assumes that an average of 2.5% of all reads represent the plastid genome, which is a common value in genome skimming experiments (Twyford and Ness, 2017; McKain et al., 2018). Although we cannot exclude that the optimal plastid genome coverage, and with it the raw sequence data needed, differs across species and datasets, we found the same result for data from two different individuals and two different assembly pipelines, indicating a potential pattern.

The results of this investigation indicate that the evenness of sequencing coverage may be an important but as of yet insufficiently recognized factor in the successful assembly of plastid genomes. Both the original and several of the capped read sets analyzed here vastly exceed the recommended level of sequencing coverage for plastid genome assembly (Twyford and Ness, 2017; McKain et al., 2018). When only considering the plastid genome reads of this uncapped read set, a sequencing depth of 8,410x and a minimum sequencing coverage of more than 1,000x in any genome position exists, indicating that the original read set of Cb01A comprises more than enough sequence information to completely assemble the plastid genome. The failure of some of the tested software tools to assemble the plastid genome is, thus, more likely associated with the unevenness than the depth of sequencing coverage. A medium but comparatively even level of sequencing coverage may be the best strategy for a successful plastid genome assembly with the tested software tools.

Our results are congruent with the findings of other investigations that report an impact of sequencing coverage on the genome assembly process (Stadermann et al., 2015; Pedersen et al., 2017) or a correlation between local extremes in sequencing coverage and assembly contig deviations (Kim et al., 2015). In general, the level of sequencing coverage is indicative for a reliable identification of sequence rearrangements and other structural variants (Sims et al., 2014; Izan et al., 2017), but the relationship between sequencing coverage and assembly reliability is not straightforward. While greater sequencing coverage typically increases the chance that rearrangement endpoints are captured and confirmed by multiple reads (Chen et al., 2009), genomic regions with exceptionally high depth of sequencing coverage have also been reported as problematic for the identification of SNPs (Li, 2014).

### 4.4. Impact of Assembly Differences on Phylogenetic Placement

The results of this investigation illustrate that a correct plastid genome assembly cannot be taken for granted without a subsequent evaluation of the assembly, even when employing dedicated software tools. Incorrect genome assemblies have the potential to affect downstream biological interpretations, such as analyses of evolutionary relationships or genetic diversity. Even if the assembly differences observed in this study only marginally affected the inferred phylogenetic position of *C. bakuense* within *Calligonum* (Figure 6 and Supplementary Figure S3), we cannot exclude the possibility that errors introduced during the assembly can lead to incorrect phylogenetic reconstructions. Plant lineages with low genetic distances between species are likely particularly sensitive to this problem (e.g., Escobari et al., 2021).

### 4.5. Recommendations for Future Studies

Given the results of this investigation, we propose three recommendations for the application of *de novo* plastid genome assembly. First, we recommend comparing the assembly results of different software tools and multiple software runs before accepting any assembly as the final genome sequence. As demonstrated here, results from different assembly software tools may vary considerably in their accuracy and repeatability. We, therefore, recommend considering only such results for subsequent analyses that are reproducible across different tools and replicate runs. This is not restricted to the four software tools tested in this investigation; there are various software applications for *de novo* genome assembly from genome skimming data, including tools specialized in circular genomes (such as plastid genomes) and general short-read assemblers. We tested three such general assemblers on the complete, unfiltered sequence dataset of *C. bakuense* in a preliminary investigation: SOAPdenovo2 (Luo et al., 2012), Platanus (Kajitani et al., 2014), and Meraculous (Chapman et al., 2011) and found that only Platanus generated assembly contigs that collectively represented either the complete plastid genome of *C. bakuense* (Cb01A) or sections of it (Cb04B). This strongly suggests that even in sequencing projects primarily targeting the nuclear genome, a separate assembly of the plastid genome with dedicated software may be required to produce reliable results. Second, we recommend capping the sequencing coverage of the input read data to an approximately even distribution along the whole genome sequence while keeping the sequencing depth within a range of 500x to 100x when conducting plastid genome assembly. While the exact relationship between sequence accuracy and both sequencing coverage and evenness is poorly understood for the assembly of plastid genomes, the results of similar investigations on bacterial genomes indicate a considerable impact of both factors (Magoc et al., 2013; Pedersen et al., 2017). More research is needed to determine the optimal balance between the depth and the evenness of sequencing coverage for reliable plastid genome assembly. Third, we recommend the release of detailed assembly and annotation information during the publication of new plastid genomes. Only by sharing a precise description of the type and succession of the software tools employed are assembly results genuinely reproducible and, ultimately, reliable (Gruening et al., 2018; Gruenstaeudl et al., 2018). The provisioning of detailed assembly and annotation information is also essential if researchers wish to re-analyze the data with new and improved methods (e.g., Gruenstaeudl, 2019). Expressly for this purpose, we release the raw sequence reads, the read datasets capped at different levels of sequencing coverage, and the raw assembly results as Supplementary Material to this investigation.

## Data Availability Statement

The datasets presented in this study can be found in online repositories. The names of the repository/repositories and accession number(s) can be found below: https://zenodo.org/record/6577786, Zenodo record 6577786; https://www.ncbi.nlm.nih.gov/sra, NCBI SRA records SRX9433946 and SRX9433941.

## Author Contributions

The study was devised by MG and KR, with participation from TB and VK. The distribution data was assessed by VK and visualized by KR. Preliminary analyses were conducted by CC and MG, and final analyzes by EG, and MG. EG performed all post-assembly finishing steps. MG performed the sequence comparisons and the phylogenetic reconstructions. KR calculated the PCoAs. The writing of the manuscript was led by MG and KR, with additional input from EG, and TB. The revision of the manuscript was organized by MG, with additional input from KR, and TB. All authors have read and approved the final version of the manuscript.

## Funding

This study was partially funded by the Volkswagen Foundation, Grant No. AZ 89 950 Developing tools for conserving the plant diversity of the South Caucasus.

## Conflict of Interest

The authors declare that the research was conducted in the absence of any commercial or financial relationships that could be construed as a potential conflict of interest.

## Publisher's Note

All claims expressed in this article are solely those of the authors and do not necessarily represent those of their affiliated organizations, or those of the publisher, the editors and the reviewers. Any product that may be evaluated in this article, or claim that may be made by its manufacturer, is not guaranteed or endorsed by the publisher.
