# Supplementary material for: Software Choice and Sequencing Coverage Can Impact Plastid Genome Assembly–A Case Study in the Narrow Endemic Calligonum bakuense
Source: Front Plant Sci. 2022 Jul 6;13:779830. doi: 10.3389/fpls.2022.779830 (PMC9296850; doi:10.3389/fpls.2022.779830)
Supplement: Supplementary file 1 [file Data_Sheet_1.pdf]

frontiers

Supplementary Material

Table S1. Overview of, and manual alignment adjustments for, the different genes, introns, and intergenic spacers extracted from the complete plastid genomes under study for phylogenetic reconstruction. Areas of unclear homology primarily represented poly-A/T microsatellites and were termed 'hotspots'.

Name	Type	Included in phylogenetic analyses	General observations	Information on hotspots and inversions
trnH-GUG	CDS	no, b/c invariable or virtually so		
trnH-GUG-psbA	IGS	yes	Cb04B_IOGA_500X exhibits many polymorphic sites	Hotspot 1: MS pos. 89-102; Hotspot 2: MS pos. 161-170
psbA	CDS	no, b/c nucleotide sequence for one or more taxa not a multiple of three		
psbA-trnK-UUU	IGS	yes		
trnK-UUU	CDS	no, b/c invariable or virtually so		
trnK-UUU_intron_1	INT	yes		
trnK-UUU_intron_2	INT	yes		
trnK-UUU-matK	IGS	no, b/c the same as trnK-UUU_intron_1 and trnK-UUU_intron_2		
matK	CDS	yes		
trnK-UUU-rps16	IGS	yes		Hotspot 1: MS pos. 78-90; Hotspot 2: MS pos. 426-439
rps16	CDS	yes		
rps16_intron	INT	yes		Hotspot 1: MS pos. 90-101
rps16-trnQ-UUG	IGS	yes	Cb04B_IOGA_repl2 exhibits various polymorphic sites; MS 2 – all assemblies from Cb01A (9) versus Cb04B differ by one T (10); the adjacent MS in pos. 1278-1298 includes the other variation in the samples	Hotspot 1: MS pos. 951-961; Hotspot 2: MS pos. 1313-1333; Inversion 1: nt 413-430, rced: TAATTCAACATCATTTTT; SNP btw. Cb01A and Cb04B
trnQ-UUG	CDS	no, b/c invariable or virtually so		
trnQ-UUG-psbK	IGS	yes	Cb04B_IOGA_repl2 exhibits various polymorphic sites; Cb04B_FaPl_rpl1 displays a 'T' in pos. 224	
psbK	CDS	yes		
psbK-psbI	IGS	yes		
psbI	CDS	yes		
psbI-trnS-GCU	IGS	yes		
trnS-GCU	CDS	no, b/c invariable or virtually so		
trnS-GCU-trnG-UCC	IGS	yes	With an alignment length of 1275 nt, this region appears unusually long	Hotspot 1: MS pos. 635-647
trnG-UCC	CDS	no, b/c invariable or virtually so		
trnG-UCC_intron	INT	yes		Hotspot 1: MS pos. 340-348
trnG-UCC-trnR-UCU	IGS	yes		
trnR-UCU	CDS	no, b/c invariable or virtually so		
trnR-UCU-atpA	IGS	yes	Cb04B FaPl_repl1 exhibits a deletion of 10nt	
atpA	CDS	yes		
atpA-atpF	IGS	yes		Hotspot 1: MS pos. 39-48
atpF	CDS	yes		
atpF_intron	INT	yes		Hotspot 1: MS pos. 270-281
atpF-atpH	IGS	yes		
atpH	CDS	yes		
atpH-atpI	IGS	yes		Hotspot 1: MS pos. 19-28; Hotspot 2: MS pos. 312-321
atpI	CDS	yes		
atpI-rps2	IGS	yes		Hotspot 1: MS pos. 28-37
rps2	CDS	yes		
rps2-rpoC2	IGS	yes		
rpoC2	CDS	yes		

Table S1. Continued.

Name	Type	Included in phylogenetic analyses	General observations	Information on hotspots and inversions
rpoC2-rpoC1	IGS	yes		
rpoC1	CDS	yes		
rpoC1_intron	INT	yes		
rpoC1-rpoB	IGS	yes		
rpoB	CDS	yes	Cb04B_IOGA_500X and Cb04B_IOGA_2000X have polymorphic sites, Cb04B_IOGA_repl1 exhibits two polymorphic sites	
rpoB-trnC-GCA	IGS	yes	Incorrect annotation in MN202595 was adjusted	
trnC-GCA	CDS	no, b/c invariable or virtually so		
trnC-GCA-petN	IGS	yes		Hotspot 1: MS pos. 375-384
petN	CDS	yes		
petN-psbM	IGS	yes		Hotspot 1: MS pos. 784-801
psbM	CDS	yes		
psbM-trnD-GUC	IGS	yes		
trnD-GUC	CDS	no, b/c invariable or virtually so		
trnD-GUC-trnY-GUA	IGS	yes		
trnY-GUA	CDS	no, b/c invariable or virtually so		
trnY-GUA-trnE-UUC	IGS	yes	Incorrect annotation in KR816224 was adjusted	
trnE-UUC	CDS	no, b/c invariable or virtually so		
trnE-UUC-trnT-GGU	IGS	yes		Hotspot 1: MS pos. 383-401; Hotspot 2: MS pos. 517-526;
trnT-GGU	CDS	no, b/c invariable or virtually so		
trnT-GGU-psbD	IGS	yes		Hotspot 1: MS pos. 220-232; Hotspot 2: MS pos. 753-764
psbD	CDS	yes		
psbD-psbC	IGS	no, b/c because flanking genes overlap		
psbC	CDS	yes		
psbC-trnS-UGA	IGS	yes		Inversion 1: KR816224 nt 199-206, rced: GGGTGAGC
trnS-UGA	CDS	no, b/c invariable or virtually so		
trnS-UGA-psbZ	IGS	yes		
psbZ	CDS	yes		
psbZ-trnG-GCC	IGS	yes		Hotspot 1: MS pos. 33-43; Hotspot 2: MS pos. 123-135;
trnG-GCC	CDS	no, b/c invariable or virtually so		
trnG-GCC-trnfM-CAU	IGS	yes		Hotspot 1: MS pos. 137-146
trnfM-CAU	CDS	no, b/c invariable or virtually so		
trnfM-CAU-rps14	IGS	yes		
rps14	CDS	yes		
rps14-psaB	IGS	yes		
psaB	CDS	yes		
psaB-psaA	IGS	yes		
psaA	CDS	yes		
psaA-pafI	IGS	yes		
pafI	CDS	no, b/c annotations not consistently present		
pafI_intron_1	INT	no, b/c annotations not consistently present		
pafI_intron_2	INT	no, b/c annotations not consistently present		
pafI-trnS-GGA	IGS	yes		Hotspot 1: MS pos. 104-118; SNP btw. Cb01A and Cb04B
trnS-GGA	CDS	no, b/c invariable or virtually so		
trnS-GGA-rps4	IGS	yes	Cb01A_IOGA_500X and Cb01A_IOGA_2000X exhibit various polymorphic sites	Inversion 1: nt 107-110 KR816224, rced: ATAG
rps4	CDS	yes		
rps4-trnT-UGU	IGS	yes	Cb01A_FaPI_repl1 and Cb01A_FaPI_repl2 exhibit gap of 47nt	

Table S1. Continued.

Name	Type	Included in phylogenetic analyses	General observations	Information on hotspots and inversions
trnT-UGU	CDS	no, b/c invariable or virtually so		
trnT-UGU-trnL-UAA	IGS	yes		Hotspot 1: MS pos. 582-594; Hotspot 2: MS pos. 653-661
trnL-UAA	CDS	no, b/c invariable or virtually so		
trnL-UAA_intron	INT	yes	Cb04B_IOGA_2000X exhibits several SNPs and an insertion of 180nt	
trnL-UAA-trnF-GAA	IGS	yes		
trnF-GAA	CDS	no, b/c invariable or virtually so		
trnF-GAA-ndhJ	IGS	yes		Inversion 1: nt 667-670 MK854996 nt 668-671 MN202609 nt 667-670 MN202610, rced: TCTT
ndhJ	CDS	yes		
ndhJ-ndhK	IGS	yes		Hotspot 1: MS pos 127-133
ndhK	CDS	yes		
ndhK-ndhC	IGS	no, b/c because flanking genes overlap	Incorrect annotation in KR816224 was adjusted	
ndhC	CDS	yes		
ndhC-trnV-UAC	IGS	yes		
trnV-UAC	CDS	no, b/c invariable or virtually so		
trnV-UAC_intron	INT	yes		
trnV-UAC-trnM-CAU	IGS	yes	Cb01A_IOGA_2000X exhibits several polymorphic sites	
trnM-CAU	CDS	no, b/c invariable or virtually so		
trnM-CAU-atpE	IGS	yes	KR816224 has an insert of length 112nt	
atpE	CDS	yes		
atpE-atpB	IGS	no, b/c because flanking genes overlap		
atpB	CDS	yes		
atpB-rbcL	IGS	yes		Hotspot 1: MS pos. 100-112; Hotspot 2: MS pos. 454-469; Hotspot 3: MS pos. 592-597
rbcL	CDS	yes		
rbcL-accD	IGS	yes		Hotspot 1: MS pos. 342-357; Hotspot 2: MS pos. 512-522
accD	CDS	yes		
accD-psaI	IGS	yes	Incorrect annotation in MN202595 was adjusted	Inversion 1: KR816224 nt 17-22, rced: GTGACA; Inversion 2: KR816224 nt 390-397, rced: TCTAAGCA
psaI	CDS	yes		
psaI-pafII	IGS	yes		
pafII	CDS	no, b/c annotations not consistently present		
pafII-cemA	IGS	yes		Hotspot 1: MS pos. 754-765
cemA	CDS	yes		
cemA-petA	IGS	yes		
petA	CDS	yes		
petA-psbJ	IGS	yes		Hotspot 1: MS pos. 927-939
psbJ	CDS	yes		
psbJ-psbL	IGS	yes		
psbL	CDS	yes	Incorrect annotation in KR816224 was adjusted	
psbL-psbF	IGS	yes	Cb01A_IOGA_500X exhibits two polymorphic sites	
psbF	CDS	yes	Cb01A_IOGA_500X exhibits five polymorphc sites	
psbF-psbE	IGS	yes		
psbE	CDS	yes		
psbE-petL	IGS	yes		Hotspot 1: MS pos. 584-595; Hotspot 2: MS pos. 1067-1077
petL	CDS	yes		
petL-petG	IGS	yes		
petG	CDS	yes		

Table S1. Continued.

Name	Type	Included in phylogenetic analyses	General observations	Information on hotspots and inversions
petG-trnW-CCA	IGS	yes		
trnW-CCA	CDS	no, b/c invariable or virtually so		
trnW-CCA-trnP-UGG	IGS	yes		
trnP-UGG	CDS	no, b/c invariable or virtually so		
trnP-UGG-psaJ	IGS	yes		Hotspot 1: MS pos. 109-118
psaJ	CDS	yes	Cb01A_NOVO_50x_seed1_partial deviates by 'KWTGY' in pos. 103-107	
psaJ-rpl33	IGS	yes		Hotspot 1: MS pos. 170-179
rpl33	CDS	yes	Cb04B_NOVO_50x_seed1_partial exhibits a 'D' in pos. 32	
rpl33-rps18	IGS	yes		
rps18	CDS	yes	Cb04B_NOVO_50x_seed1_partial exhibits three polymorphic sites	
rps18-rpl20	IGS	yes		
rpl20	CDS	yes		
rpl20-rps12	IGS	no, b/c rps12 is trans-spliced and its bioinformatic extraction prone to error		
rps12	CDS	no, b/c rps12 is trans-spliced and its bioinformatic extraction prone to error		
rps12-rps12	IGS	no, b/c rps12 is trans-spliced and its bioinformatic extraction prone to error		
rps12	CDS	no, b/c rps12 is trans-spliced and its bioinformatic extraction prone to error		
rps12-clpP	IGS	no, b/c rps12 is trans-spliced and its bioinformatic extraction prone to error		
clpP	CDS	yes	Cb04b_NOVO_50X_seed1_partial differs by K in pos. 275, R in pos. 628 by a 'TWK' insertion at the end (gap in others)	
clpP_intron_1	INT	yes	Cb04B_NOVO_50X_seed1_partial exhibits several polymorphic sites; CB04B_IOGA_2000X exhibits two inserted Ns	Hotspot 1: MS pos. 61-69; Hotspot 2: MS pos. 785-798
clpP_intron_2	INT	yes		Hotspot 1: MS pos. 488-499
clpP-psbB	IGS	yes		Inversion 1: RE816224 nt 85-99, rced: CATATATGACAAAG
psbB	CDS	yes	Cb04B_NOVO_50x_seed1_partial differs by two polymorphic sites (pos. 1300-1312; 'YGTGCTACTTTGM') and four polymorphic sites (pos. 136-143; 'KHTYCCTY')	
psbB-psbT	IGS	yes		
psbT	CDS	yes		
psbT-psbN	IGS	yes	INV in various genomes: MK854997, MN202595, MN202596, MN202597, MN202599, MN202600, MN202601, MN202602, MN202603, MN202604, MN202608, MN202609, MN202610, MN202611, MN202612, MN202613	Inversion 1: MK854997 MN202595 MN202596 MN202597 MN202599 MN202600 MN202601 MN202602 MN202603 MN202604 MN202608 MN202609 MN202610 MN202611 MN202612 MN202613 nt 41-47, rced: TGAATGC
psbN	CDS	yes		
psbN-psbH	IGS	yes		
psbH	CDS	yes		
psbH-petB	IGS	yes		

Table S1. Continued.

Name	Type	Included in phylogenetic analyses	General observations	Information on hotspots and inversions
petB	CDS	no, b/c various GenBank records exhibit incorrect annotations		
petB_intron	INT	yes		
petB-petD	IGS	yes		
petD	CDS	yes		
petD_intron	INT	yes		
petD-rpoA	IGS	yes		
rpoA	CDS	yes		
rpoA-rps11	IGS	yes		
rps11	CDS	yes	Cb04B_NOVO_50X_seed1_partial exhibits one polymorphic site	
rps11-rpl36	IGS	yes	Cb01A_NOVO_500X_seed2 exhibits a 'W' in pos. x	Hotspot 1: MS pos. 77-85
rpl36	CDS	yes		
rpl36-infA	IGS	yes	Incorrect annotation in KR816224 was adjusted	
infA	CDS	yes		
infA-rps8	IGS	yes		
rps8	CDS	yes		
rps8-rpl14	IGS	yes		
rpl14	CDS	yes		
rpl14-rpl16	IGS	yes		
rpl16	CDS	no, b/c nucleotide sequence for one or more taxa not a multiple of three		
rpl16_intron	INT	yes		
rpl16-rps3	IGS	yes		
rps3	CDS	yes	Cb04B_NOVO_50x_seed1_partial exhibits two polymorphic sites	
rps3-rpl22	IGS	no, b/c because flanking genes overlap		
rpl22	CDS	yes		
rpl22-rps19	IGS	yes		
rps19	CDS	yes		
rps19-rpl2	IGS	yes		Hotspot 1: MS pos. 16-27
rpl2	CDS	no, b/c various GenBank records exhibit incorrect annotations		
rpl2_intron	INT	yes	Cb01A_IOGA_500X and Cb01A_IOGA_2000X have insertions of 219nt and several polymorphic sites and SNPs; Cb01A_IOGA_2000X has two additional insertions (of 146nt and 432nt, respectively) and several additional SNPs and indels; Cb04B_IOGA_500X has similar insertions to those of Cb01A_IOGA_2000X (e.g., 432nt and 62nt); Cb01A_FaPl_repl1 and Cb01A_FaPl_repl2 exhibits various SNPs and indels; Cb04B_IOGA_2000X, Cb04b_IOGA_repl2, and Cb04B_NOVO_50x_seed1_partial exhibit several polymorphic sites	
rpl2-rpl23	IGS	yes	Cb04B_IOGA_500X exhibits two polymorphic sites	
rpl23	CDS	no, b/c nucleotide sequence for one or more taxa not a multiple of three		
rpl23-trnI-CAU	IGS	no, b/c both annotations of flanking regions unreliable		
trnI-CAU	CDS	no, b/c various GenBank records exhibit incorrect annotations		

Table S1. Continued.

Name	Type	Included in phylogenetic analyses	General observations	Information on hotspots and inversions
trnI-CAU-ycf2	IGS	no, b/c both annotations of flanking regions unreliable		
ycf2	CDS	no, b/c nucleotide sequence for one or more taxa not a multiple of three		
ycf2-trnL-CAA	IGS	no, b/c both annotations of flanking regions unreliable		
trnL-CAA	CDS	no, b/c invariable or virtually so		
trnL-CAA-ndhB	IGS	yes	Incorrect annotation in KR816224 was adjusted	
ndhB	CDS	yes		
ndhB_intron	INT	yes	CB04B_IOGA_2000X exhibits a large (133nt), unique insertion	
ndhB-rps7	IGS	yes		
rps7	CDS	yes	Cb01A_IOGA_500X and Cb01A_IOGA_2000X exhibit various polymorphic sites	
rps7-trnV-GAC	IGS	yes	Cb01A_IOGA_500X and Cb01A_IOGA_2000X exhibit various polymorphic sites; Cb01A_FaPl_repl2 has an additional 'c' in MS1	
trnV-GAC	CDS	no, b/c invariable or virtually so		
trnV-GAC-rrn16	IGS	yes	many deviating substitutions and indels in Cb04B_IOGA_repl1, Cb04B_IOGA_repl2, Cb04B_IOGA_2000X, Cb04B_IOGA_500X, Cb01B_IOGA_repl2, Cb01B_IOGA_2000X, Cb01B_IOGA_500X; some of these substitutions and indels similar to those of KR816224	
rrn16	CDS	no, b/c invariable or virtually so		
rrn16-trnI-GAU	IGS	yes		
trnI-GAU	CDS	no, b/c invariable or virtually so		
trnI-GAU_intron	INT	yes	Cb04b_IOGA_repl2 exhibits several polymorphic sites	
trnI-GAU-trnA-UGC	IGS	yes		
trnA-UGC	CDS	no, b/c invariable or virtually so		
trnA-UGC_intron	INT	yes		
trnA-UGC-rrn23	IGS	yes		
rrn23	CDS	no, b/c invariable or virtually so		
rrn23-rrn45	IGS	yes		
rrn45	CDS	no, b/c invariable or virtually so		
rrn45-rrn5	IGS	yes	Incorrect annotation in KR816224 was adjusted	
rrn5	CDS	no, b/c invariable or virtually so		
rrn5-trnR-ACG	IGS	yes	Incorrect annotation in KR816224 was adjusted	
trnR-ACG	CDS	no, b/c invariable or virtually so		
trnR-ACG-trnN-GUU	IGS	yes	Cb04B_IOGA_2000X deviates by several substitutions and exhibits an insertion of 171nt; Cb01A_FaPl_repl2 exhibits an inversion (motif 'cttt') that is shared with KR816224 and MN202602; incorrect annotation in KR816224 was adjusted	
trnN-GUU	CDS	no, b/c invariable or virtually so		
trnN-GUU-ycf1	IGS	yes	Cb01a_FaPl_repl2 exhibits various SNPs and insertions	
ycf1	CDS	no, b/c nucleotide sequence for one or more taxa not a multiple of three		
ycf1-ndhF	CDS	yes	Cb04B_NOVO_1000X_seed1 and Cb04B_IOGA_2000X exhibit unique insertions	

Table S1. Continued.

Name	Type	Included in phylogenetic analyses	General observations	Information on hotspots and inversions
ndhF	CDS	no, b/c nucleotide sequence for one or more taxa not a multiple of three		
ndhF-rpl32	IGS	yes		Hotspot 1: MS pos. 473-482; Hotspot 2: MS pos. 544-554; SNP btw. Cb01A and Cb04B
rpl32	CDS	yes		
rpl32-trnL-UAG	IGS	yes	Cb01A_NOVO_2000X_seed1 is the only assembly that has one 'T' less in in HS2 (MS)	Hotspot 1: MS pos. 71-82; Hotspot 2: MS pos. 133-142; Hotspot 3: MS pos. 259-272
trnL-UAG	CDS	no, b/c invariable or virtually so		
trnL-UAG-ccsA	IGS	yes		
ccsA	CDS	yes		
ccsA-ndhD	IGS	yes		
ndhD	CDS	yes	Various assemblies exhibit gaps at 5' end, among them in many cases the additional sequence 'GTGTATTTTGTCTTTACC' upstream of the GenBank records	
ndhD-psaC	IGS	yes	Various assemblies exhibit insertions at 5' end	
psaC	CDS	yes		
psaC-ndhE	IGS	yes		
ndhE	CDS	yes	Annotation was corrected in KR816224, MN202603, and MN202613 (i.e., removal of three superfluous ATG at the beginning)	
ndhE-ndhG	IGS	yes		
ndhG	CDS	yes		
ndhG-ndhI	IGS	yes		
ndhI	CDS	yes		
ndhI-ndhA	IGS	yes		
ndhA	CDS	yes		
ndhA_intron	INT	yes	Cb01A_NOVO_2000X_seed1 and Cb04B_NOVO_2000X_seed1 exhibit the same gap in each of the two assemblies in a minisatellite-like AT-rich section of the genome	
ndhA-ndhH	IGS	yes		
ndhH	CDS	no, b/c nucleotide sequence for one or more taxa not a multiple of three		
ndhH-rps15	IGS	yes		
rps15	CDS	yes	Cb01A_NOVO_2000X_seed2 is deviating with a 'T' in pos. 21	
rps15-ycf1	IGS	yes	Several assemblies exhibit indels; incorrect annotation in KR816224 was adjusted	

Table S2. Numeric comparison of the plastid genome assemblies of individual Cb01A as generated by different assembly software, levels of sequencing coverage, seed sequences, and run replicates. Numbers in parentheses indicate numbers as duplicated in the IR.

Treatment	GetO repl1	GetO repl2	GetO 2000X	GetO 1000X partial	GetO 500X	GetO 250X	GetO 100X	GetO 50X
Genome size (bp)	162,128	162,128	162,128	62,295	162,128	162,128	162,128	160,238
LSC length (bp)	87,689	87,689	87,689	incomplete	87,689	87,689	87,689	89,61
SSC length (bp)	13,387	13,387	13,387	incomplete	13,387	13,387	13,387	13,387
IR length (bp)	30,526	30,526	30,526	30,526	30,526	30,526	30,526	28,61
Number of genes	113	113	113	39	113	109	109	109
Number of protein-coding genes	79 (7)	79 (7)	79 (7)	21 (0)	79 (7)	79 (7)	79 (7)	75 (4)
Number of tRNA genes	30 (7)	30 (7)	30 (7)	14 (0)	30 (7)	30 (7)	30 (7)	30 (7)
Number of rRNA genes	4 (4)	4 (4)	4 (4)	4 (0)	4 (4)	4 (4)	4 (4)	4 (4)
Number of genes with one intron (two introns)	15 (3)	15 (3)	15 (3)	9 (0)	15 (3)	15 (3)	15 (3)	12 (2)
Proportion of coding to non-coding regions	0.70	0.71	0.71	0.71	0.71	0.71	0.71	0.69
Average gene density (genes/kb)	0.81	0.81	0.81	0.63	0.81	0.81	0.81	0.77
GC content (%)	37.5	37.5	37.5	38.1	37.5	37.5	37.5	37.5

Treatment	NOVO repl1 seed1	NOVO repl1 seed2	NOVO repl2 seed1	NOVO repl2 seed2	NOVO 2000X seed1	NOVO 2000X seed2
Genome size (bp)	170,009	170,003	170,099	170,106	162,263	162,128
LSC length (bp)	67,504	42,1	47,218	32,997	87,689	87,689
SSC length (bp)	13,387	13,387	13,387	13,387	13,336	13,387
IR length (bp)	44,559	57,258	54,747	61,861	30,526	30,526
Number of genes	108	96	99	95	113	113
Number of protein-coding genes	76 (23)	68 (38)	71 (34)	67 (38)	79 (7)	79 (7)
Number of tRNA genes	28 (7)	24 (9)	24 (9)	24 (9)	30 (7)	30 (7)
Number of rRNA genes	4 (4)	4 (4)	4 (4)	4 (4)	4 (4)	4 (4)
Number of genes with one intron (two introns)	13 (3)	11 (3)	12 (3)	10 (3)	15 (3)	15 (3)
Proportion of coding to non-coding regions	0.69	0.68	0.71	0.68	0.71	0.71
Average gene density (genes/kb)	0.84	0.86	0.86	0.86	0.81	0.81
GC content (%)	37.5	37.5	37.5	37.5	37.5	37.5

Treatment	NOVO 1000X	NOVO 500X seed1	NOVO 500X seed2	NOVO 250X	NOVO 100X	NOVO 50X partial
Genome size (bp)	162,128	162,151	162,128	162,128	162,128	117,861
LSC length (bp)	87,689	87,689	87,689	87,689	87,689	87,335
SSC length (bp)	13,387	13,387	13,387	13,387	13,387	incomplete
IR length (bp)	30,526	30,526	30,526	30,516	30,526	30,526
Number of genes	109	113	113	109	109	96
Number of protein-coding genes	79 (7)	79 (7)	79 (7)	79 (7)	79 (7)	64 (0)
Number of tRNA genes	30 (7)	30 (7)	30 (7)	30 (7)	30 (7)	28 (0)
Number of rRNA genes	4 (4)	4 (4)	4 (4)	4 (4)	4 (4)	4 (0)
Number of genes with one intron (two introns)	15 (3)	15 (3)	15 (3)	15 (3)	15 (3)	11 (2)
Proportion of coding to non-coding regions	0.71	0.71	0.71	0.71	0.71	0.68
Average gene density (genes/kb)	0.81	0.81	0.81	0.81	0.81	0.81
GC content (%)	37.5	37.5	37.5	37.5	37.5	37.1

Table S2. Continued.

Treatment	FaPl repl1	FaPl repl2	FaPl 2000X	FaPl 500X	IOGA repl1	IOGA repl2	IOGA 2000X	IOGA 500X
Genome size (bp)	162,093	162,173	162,128	163,006	163,538	164,532	165,56	163,478
LSC length (bp)	87,642	87,642	87,689	87,689	87,957	87,957	88,194	88,394
SSC length (bp)	13,387	13,387	13,387	13,387	13,387	13,387	13,388	13,388
IR length (bp)	30,532	30,572	30,526	30,526	31,097	31,594	31,989	30,848
Number of genes	111	113	113	113	109	114	113	112
Number of protein-coding genes	78 (6)	79 (7)	79 (7)	79 (9)	76 (5)	79 (9)	79 (7)	79 (7)
Number of tRNA genes	30 (7)	30 (7)	30 (7)	30 (7)	30 (7)	30 (7)	30 (6)	29 (7)
Number of rRNA genes	3 (3)	4 (4)	4 (4)	4 (4)	3 (3)	5 (3)	4 (4)	4 (4)
Number of genes with one intron (two introns)	15 (3)	15 (3)	15 (3)	15 (3)	15 (3)	15 (3)	15 (3)	15 (3)
Proportion of coding to non-coding regions	0.68	0.71	0.71	0.71	0.59	0.71	0.69	0.71
Average gene density (genes/kb)	0.78	0.81	0.81	0.82	0.76	0.81	0.79	0.80
GC content (%)	37.4	37.3	37.5	37.5	37.5	37.5	37.3	37.3

Table S3. Numeric comparison of the plastid genome assemblies of individual Cb04B as generated by different assembly software, levels of sequencing coverage, seed sequences, and run replicates. Numbers in parentheses indicate numbers as duplicated in the IR.

Treatment	GetO repl1	GetO repl2	GetO 2000X	GetO 1000X partial	GetO 500X	GetO 250X	GetO 100X	GetO 50X
Genome size (bp)	162,129	162,129	162,129	67,160	162,129	162,129	162,129	162,150
LSC length (bp)	87,689	87,689	87,689	incomplete	87,689	87,689	87,689	87,689
SSC length (bp)	13,388	13,388	13,388	incomplete	13,388	13,388	13,388	13,388
IR length (bp)	30,526	30,526	30,526	30,526	30,526	30,526	30,526	30,526
Number of genes	113	113	113	44	113	113	113	113
Number of protein-coding genes	79 (7)	79 (7)	79 (7)	22 (0)	79 (7)	79 (7)	79 (7)	79 (7)
Number of tRNA genes	30 (7)	30 (7)	30 (7)	18 (0)	30 (7)	30 (7)	30 (7)	30 (7)
Number of rRNA genes	4 (4)	4 (4)	4 (4)	4 (0)	4 (4)	4 (4)	4 (4)	4 (4)
Number of genes with one intron (two introns)	15 (3)	15 (3)	15 (3)	9 (0)	15 (3)	15 (3)	15 (3)	15 (3)
Proportion of coding to non-coding regions	0.70	0.71	0.71	0.68	0.71	0.71	0.71	0.71
Average gene density (genes/kb)	0.81	0.81	0.81	0.66	0.81	0.81	0.81	0.81
GC content (%)	37.5	37.5	37.5	38.0	37.5	37.5	37.5	37.5

Treatment	NOVO repl1 seed1	NOVO repl1 seed2	NOVO repl2 seed1	NOVO repl2 seed2	NOVO 2000X seed1	NOVO 2000X seed2
Genome size (bp)	162,129	162,129	162,129	162,129	162,078	162,129
LSC length (bp)	87,689	87,789	87,689	87,689	87,689	87,689
SSC length (bp)	13,388	13,388	13,388	13,388	13,337	13,388
IR length (bp)	30,526	30,476	30,526	30,526	30,526	30,526
Number of genes	113	113	113	114	113	113
Number of protein-coding genes	79 (7)	79 (7)	79 (7)	80 (6)	79 (7)	79 (7)
Number of tRNA genes	30 (7)	30 (7)	30 (7)	30 (7)	30 (7)	30 (7)
Number of rRNA genes	4 (4)	4 (4)	4 (4)	4 (4)	4 (4)	4 (4)
Number of genes with one intron (two introns)	15 (3)	15 (3)	15 (3)	15 (4)	15 (3)	15 (3)
Proportion of coding to non-coding regions	0.70	0.70	0.71	0.71	0.71	0.71
Average gene density (genes/kb)	0.81	0.81	0.81	0.81	0.81	0.81
GC content (%)	37.5	37.5	37.5	37.5	37.5	37.5

Treatment	NOVO 1000X	NOVO 500X seed1	NOVO 500X seed2	NOVO 250X	NOVO 100X partial	NOVO 50X partial
Genome size (bp)	162,129	162,129	162,129	162,129	112,054	75,891
LSC length (bp)	87,689	87,689	87,689	87,739	incomplete	incomplete
SSC length (bp)	13,396	13,388	13,388	13,388	13,388	13,388
IR length (bp)	30,526	30,526	30,526	30,476	30,526	30,526
Number of genes	112	112	113	110	70	46
Number of protein-coding genes	78 (8)	78 (8)	79 (7)	79 (7)	52 (6)	34 (1)
Number of tRNA genes	30 (7)	30 (7)	30 (7)	30 (7)	14 (7)	8 (4)
Number of rRNA genes	4 (4)	4 (4)	4 (4)	4 (4)	4 (4)	4 (4)
Number of genes with one intron (two introns)	14 (3)	14 (3)	15 (3)	15 (3)	7 (1)	5 (1)
Proportion of coding to non-coding regions	0.71	0.71	0.71	0.71	0.72	0.77
Average gene density (genes/kb)	0.81	0.81	0.81	0.81	0.78	0.72
GC content (%)	37.5	37.5	37.5	37.5	38.3	39.0

Table S3. Continued.

Treatment	FaPl repl1	FaPl repl2	FaPl 2000X	FaPl 500X	IOGA repl1	IOGA repl2	IOGA 2000X	IOGA 500X
Genome size (bp)	175,272	175,512	162,129	163,292	166,405	167,837	163,822	165,285
LSC length (bp)	74,285	74,295	87,689	87,689	88,717	88,737	88,204	89,094
SSC length (bp)	13,333	13,333	13,388	13,785	13,388	13,39	13,388	13,388
IR length (bp)	43,827	43,942	30,526	30,526	32,15	32,855	31,115	31,406
Number of genes	113	114	113	113	112	113	114	113
Number of protein-coding genes	79 (23)	80 (22)	79 (7)	79 (10)	78 (5)	79 (8)	79 (7)	79 (7)
Number of tRNA genes	30 (7)	30 (7)	30 (7)	30 (7)	30 (7)	30 (6)	31 (6)	30 (7)
Number of rRNA genes	4 (4)	4 (4)	4 (4)	4 (4)	4 (4)	4 (4)	4 (4)	4 (4)
Number of genes with one intron (two introns)	15 (3)	15 (3)	15 (3)	15 (3)	15 (3)	15 (3)	15 (3)	15 (3)
Proportion of coding to non-coding regions	0.70	0.72	0.71	0.71	0.66	0.69	0.71	0.71
Average gene density (genes/kb)	0.84	0.84	0.81	0.82	0.77	0.78	0.80	0.79
GC content (%)	37.4	37.3	37.5	37.5	37.5	37.4	37.3	37.1

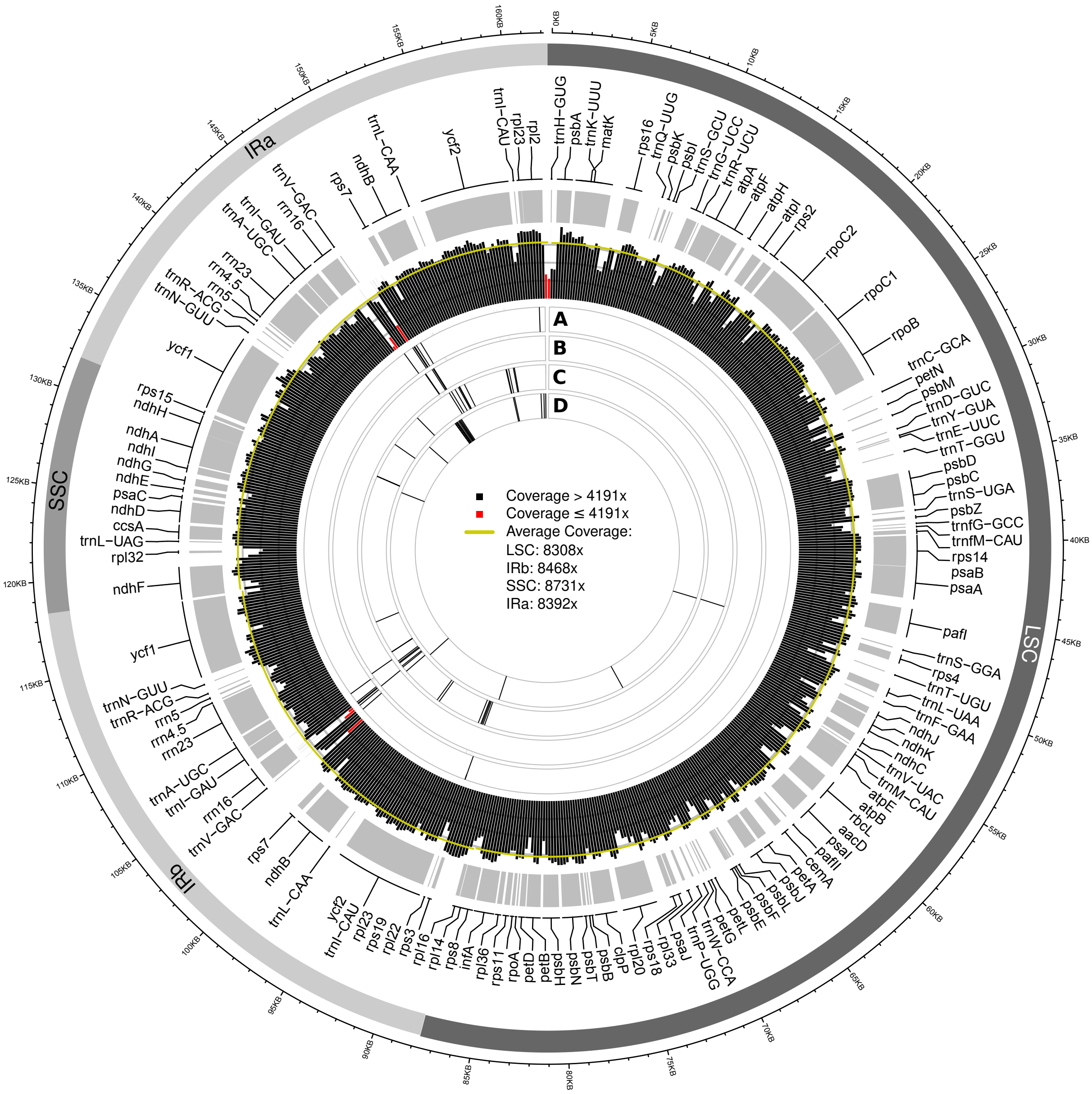

Figure S1. Visualization of the sequencing coverage of the plastid genome of Cb01A assembled with IOGA relative to the location of SNPs for assemblies generated with the same assembly software but under different levels of sequencing coverage. All colors, references, and abbreviations are as in Figure 5 of the main text.

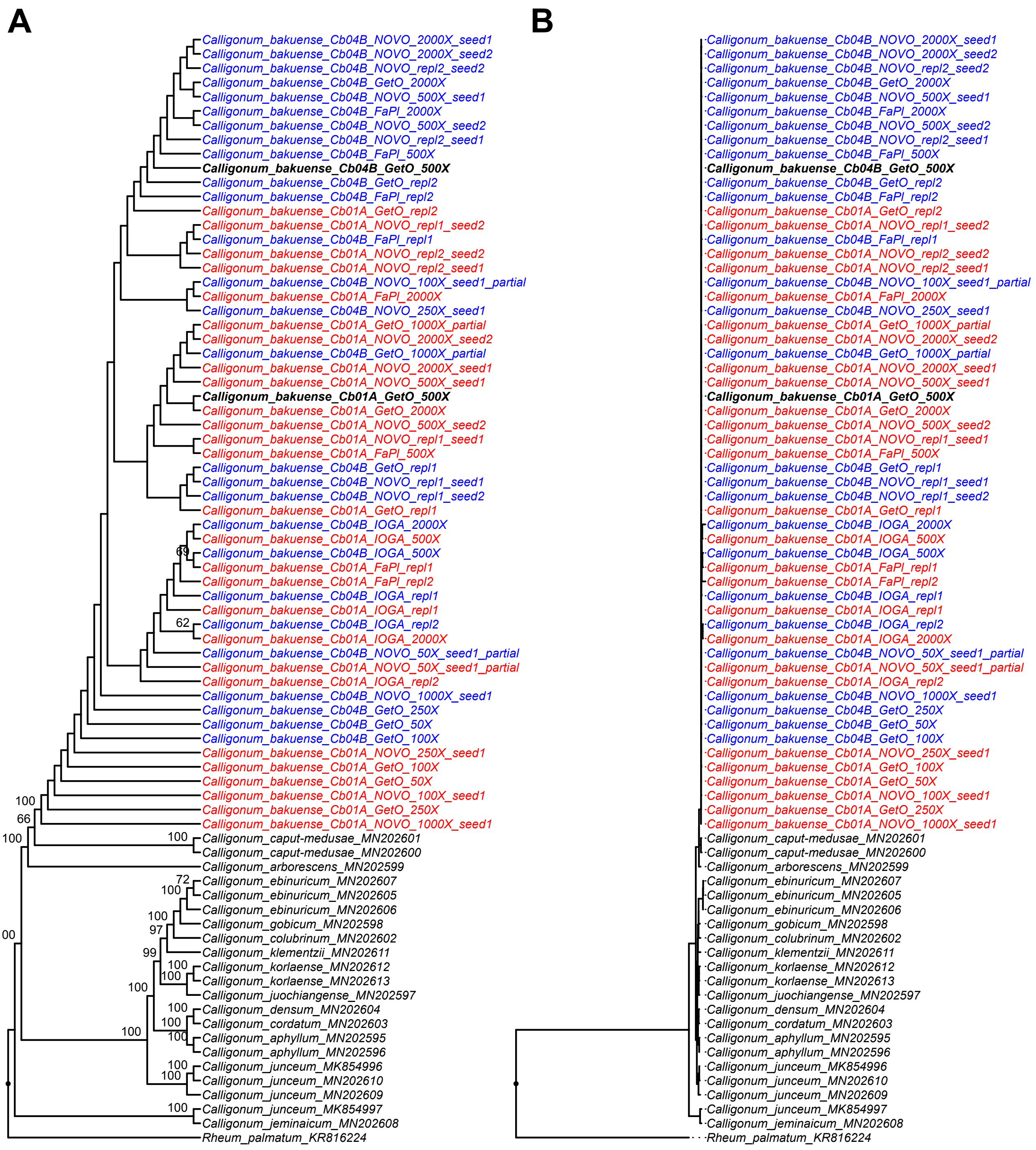

Figure S2. The phylogenetic position of the plastid genome assemblies of *C. bakuense* as generated with different assembly software, levels of sequencing coverage, seed sequences, and run replicates in relation to other species of *Calligonum*. The matrix employed for phylogenetic reconstruction comprises both coding and non-coding regions of the plastid genomes but insertions and deletions in the matrix are uncoded. Assemblies of Cb01A are highlighted in red, those of Cb04B in blue. The two final plastid genome sequences of *C. bakuense* are highlighted in bold. The displayed phylogenetic tree represents the best tree inferred under ML, visualized as **(A)** cladogram with bootstrap support (>50%, above branches) and **(B)** the corresponding phylogram with exact branch lengths.

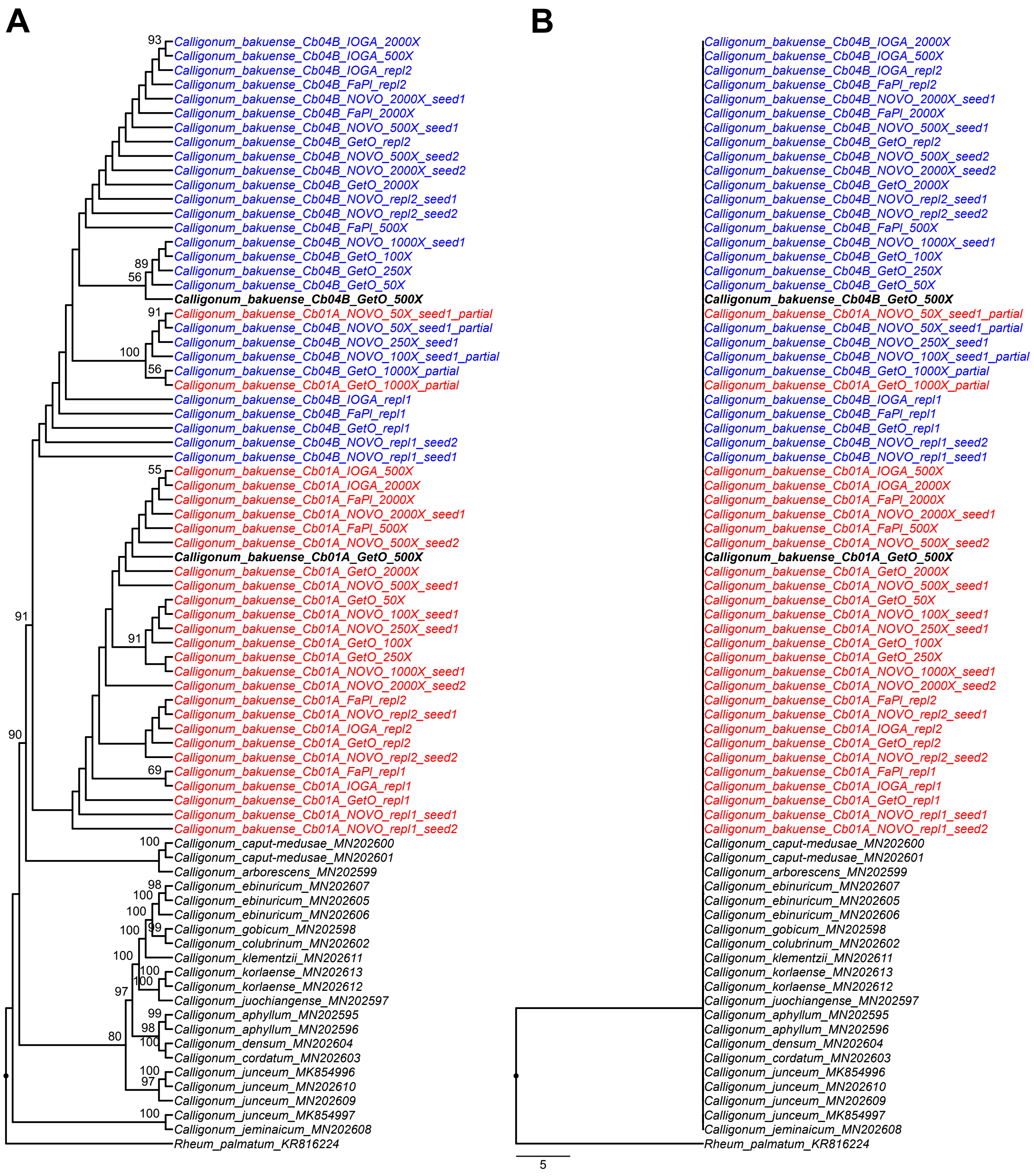

Figure S3. The phylogenetic position of the plastid genome assemblies of *C. bakuense* as generated with different assembly software, levels of sequencing coverage, seed sequences, and run replicates in relation to other species of *Calligonum*, with insertions and deletions in the underlying matrix coded according to the simple indel coding scheme. Assemblies of Cb01A are highlighted in red, those of Cb04B in blue. The two final plastid genome sequences of *C. bakuense* are highlighted in bold. The displayed phylogenetic tree represents the best tree inferred under ML, visualized as **(A)** cladogram with bootstrap support (>50%, above branches) and **(B)** the corresponding phylogram with exact branch lengths.
